# Multidimensional profiling of human T cells reveals high CD38 expression, marking recent thymic emigrants and age-related naive T cell remodeling

**DOI:** 10.1016/j.immuni.2024.08.019

**Published:** 2024-09-24

**Authors:** Pavla Bohacova, Marina Terekhova, Petr Tsurinov, Riley Mullins, Kamila Husarcikova, Irina Shchukina, Alina Ulezko Antonova, Barbora Echalar, Jan Kossl, Adam Saidu, Thomas Francis, Chelsea Mannie, Laura Arthur, Stephen D.R. Harridge, Daniel Kreisel, Philip A. Mudd, Angela M. Taylor, Coleen A. McNamara, Marina Cella, Sidharth V. Puram, Theo van den Broek, Femke van Wijk, Pirooz Eghtesady, Maxim N. Artyomov

**Affiliations:** 1 Department of Pathology and Immunology, Washington University School of Medicine, St. Louis, MO 63110, USA; 2 JetBrains Research, Paphos 8021, Cyprus; 3 Department of Otolaryngology-Head and Neck Surgery, Washington University School of Medicine, St. Louis, MO 63110, USA; 4 Department of Genetics, Washington University School of Medicine, St. Louis, MO 63110, USA; 5 Department of Emergency Medicine, Washington University School of Medicine, St. Louis, MO 63110, USA; 6 Centre for Human and Applied Physiological Sciences, School of Basic and Medical Biosciences, Faculty of Life Sciences & Medicine, King’s College London, London SE1 1UL, UK; 7 Division of Cardiothoracic Surgery, Washington University School of Medicine, St. Louis, MO 63110, USA; 8 Department of Surgery, Washington University School of Medicine, St. Louis, MO 63110, USA; 9 The Andrew M. and Jane M. Bursky Center for Human Immunology & Immunotherapy Programs, Washington University School of Medicine, St. Louis, MO 63110, USA; 10 Center for Vaccines and Immunity to Microbial Pathogens, Washington University School of Medicine, St. Louis, MO 63110, USA; 11 Department of Medicine, Cardiovascular Division, University of Virginia, Charlottesville, VA 22903, USA; 12 Beirne B. Carter Center for Immunology Research, University of Virginia, Charlottesville, VA 22903, USA; 13 Rob Ebert and Greg Stubblefield Head and Neck Tumor Center at Siteman Cancer Center, St. Louis, MO 63110, USA; 14 Center for Translational Immunology, University Medical Centre Utrecht, Utrecht University, Utrecht 3584CX, the Netherlands; 15 These authors contributed equally; 16 Lead contact

## Abstract

Thymic involution is a key factor in human immune aging, leading to reduced thymic output and a decline in recent thymic emigrant (RTE) naive T cells in circulation. Currently, the precise definition of human RTEs and their corresponding cell surface markers lacks clarity. Analysis of single-cell RNA-seq/ATAC-seq data distinguished RTEs by the expression of SOX4, IKZF2, and TOX and CD38 protein, whereby surface CD38^hi^ expression universally identified CD8^+^ and CD4^+^ RTEs. We further determined the dynamics of RTEs and mature cells in a cohort of 158 individuals, including age-associated transcriptional reprogramming and shifts in cytokine production. Spectral cytometry profiling revealed two axes of aging common to naive CD8^+^ and CD4^+^ T cells: (1) a decrease in CD38^++^ cells (RTEs) and (2) an increase in CXCR3^hi^ cells. Identification of RTEs enables direct assessment of thymic health. Furthermore, resolving the dynamics of naive T cell remodeling yields insight into vaccination and infection responsiveness throughout aging.

## In brief

Identifying human T cells that have recently emigrated from the thymus remains problematic. Here, Bohacova et al. categorize recent thymic emigrants with high CD38 expression as their universal surface marker. Additionally, they explore the transcriptional and epigenetic landscape of naive CD8^+^ and CD4^+^ T cells, revealing shared axes of aging.

## Graphical Abstract

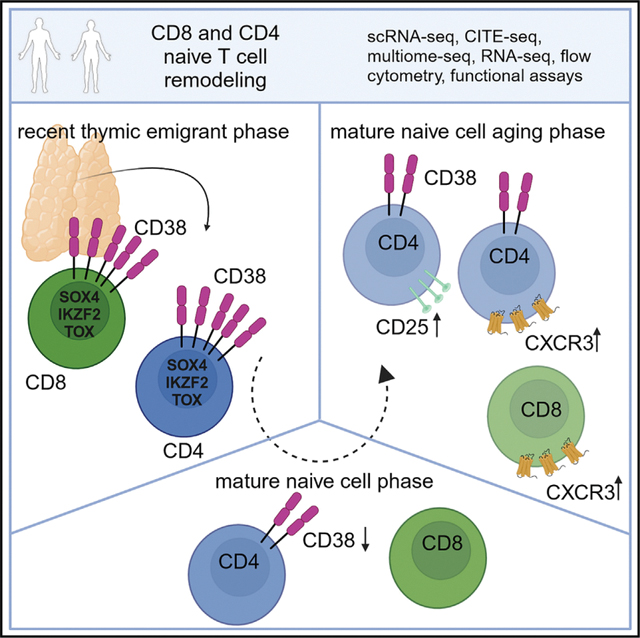

## INTRODUCTION

The most apparent physiological change associated with immune aging is thymic involution,^1^ which leads to a diminished generation of naive T cells.^2–5^ In humans, the thymic output is not always directly reflected in the quantity of naive cells, e.g., naive CD4^+^ T cells do not decline considerably with age.^6–9^ Instead, thymic output can be characterized by the presence of recent thymic emigrants (RTEs) circulating in the bloodstream.^10–12^ Therefore, the ability to examine levels of RTEs could provide a direct measure of immune aging. However, in contrast to animal models that allow genetic and/or surgical interventions, accurate identification of RTEs in humans has proven to be challenging.^10,13^

Several strategies exist for identifying RTEs in humans. First, the enrichment of a T cell receptor (TCR) excision circle (TREC) is commonly considered to define RTEs in both CD8^+^ and CD4^+^ T cells.^12,13^ However, there are some major conceptual and practical limitations to using TREC as a marker of RTEs. First, RTEs may lack TREC due to dilution from late intrathymic proliferation at the single positive (SP) thymocyte stage.^14,15^ Second, since TREC only gets diluted inside the T cell pool, one can observe TREC^+^ cells even decades post-thymectomy.^12^ Lastly, the inherent nature of TREC detection prohibits cell sorting, limiting its practical utilization as a marker.^10^ Therefore, while TREC enrichment is valuable for comparing subpopulations regarding thymic egress, it cannot effectively serve as an RTE marker.

Several surface markers are considered to identify RTE cells. Surface CD31 expression is suggested as an RTE marker of naive CD4^+^ T cells based on the enrichment in TREC signal.^16^ However, CD31^+^ naive CD4^+^ T cells are still highly present in the patients after thymectomy,^17^ suggesting that CD31 expression might be too broad to designate these cells as RTEs accurately. Alternatively, later studies propose that the most immature fraction of CD4^+^ RTEs might be enriched in PTK7.^18,19^ The PTK7^+^ cells appear to define the subset of RTEs that most recently egressed from the thymus, as PTK7 expression is lost in 2–3 proliferative cycles.^17^ Overall, currently, there are no reliable surface markers associated with RTEs in the naive CD8^+^ T cell pool, and there is only limited understanding of RTEs within naive CD4^+^ T cells.

The challenge of identifying reliable markers is closely connected to the broader difficulty of impartially defining the subpopulation of RTEs. In this work, we unbiasedly identify a transcriptionally and epigenetically distinct subpopulation of CD8^+^ and CD4^+^ RTEs as *SOX4*^+^*IKZF2*^+^*TOX*^+^ naive T cells, marked by high expression of surface CD38. This reveals CD38 as a universal surface marker for RTEs, enabling their phenotypic and functional characterization.

## RESULTS

### SOX4^+^ naive CD8^+^ and CD4^+^ T cells are a transcriptionally distinct population that declines with age

**Figure 1.**
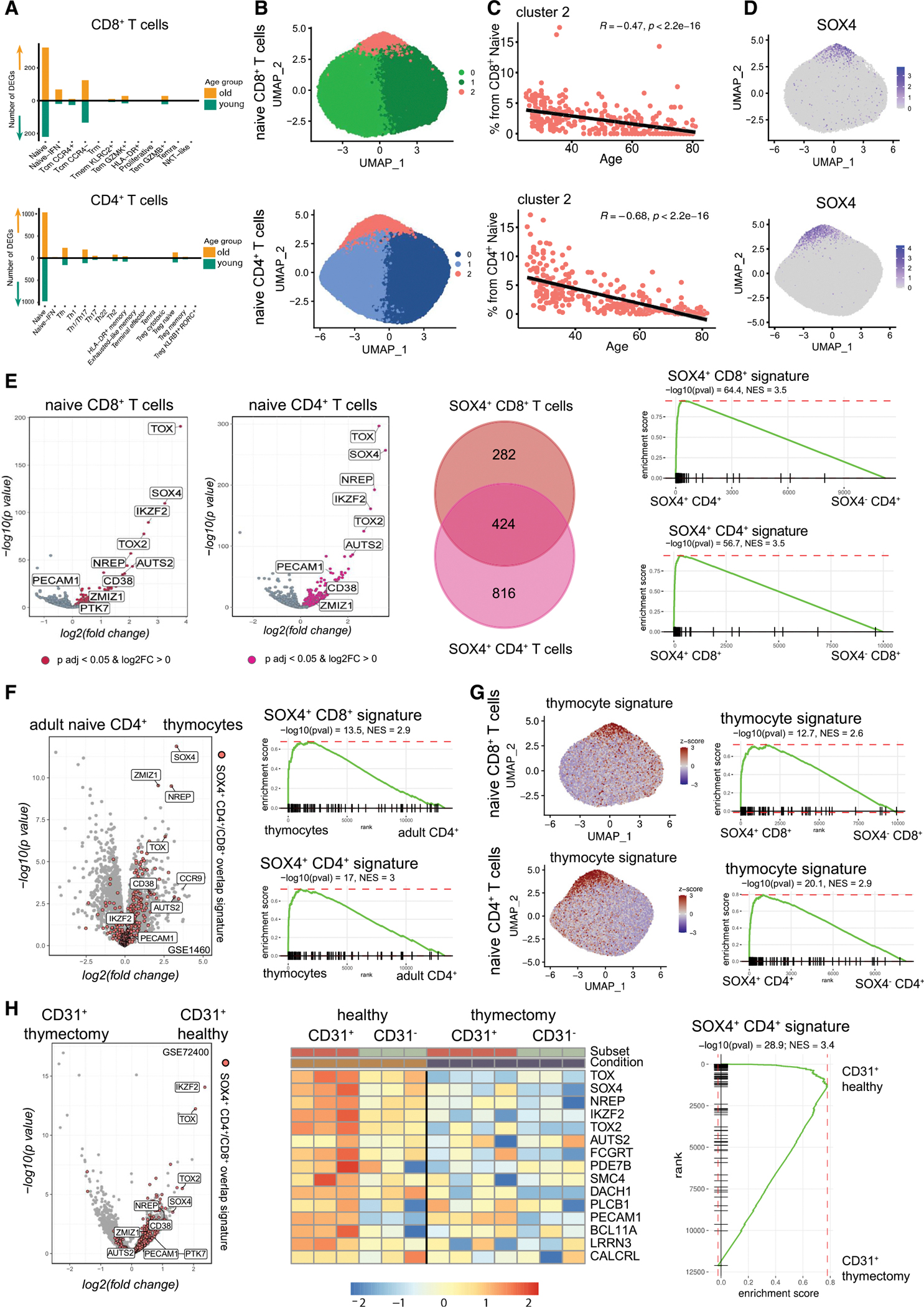
Characterization of SOX4^+^ clusters of naive T cells (A) Bar plots showing a number of differentially expressed genes between old (>64 years old) vs. young (<35 years old) in T cell clusters. (B) Uniform manifold and approximation projection (UMAP) plot of naive CD8^+^ and CD4^+^ T cells from the ABF300 study, colored by cluster. (C) Scatterplot showing the percentage of cluster 2 from a total number of naive T cells against age. Black line represents the best-fitted linear regression, with the shading showing the 95% confidence intervals. R represents the Pearson correlation coefficient. (D) UMAP plots showing normalized expression for *SOX4* gene in naive T cells. (E) Volcano plot for comparison of SOX4^+^ vs. SOX4^−^ clusters in naive cells (left). Venn diagram showing differentially expressed gene overlap (*p*.adj < 0.05 and log_2_(fold change [FC]) > 0) between SOX4^+^ clusters in naive CD8^+^ and CD4^+^ T cells (middle). GSEA plots of the top 100 differentially expressed genes in CD4^+^ SOX4^+^ cluster enriched to CD8^+^ SOX4^+^ cluster and vice versa (right). (F) Volcano plot for comparison of thymocytes vs. adult naive CD4^+^ T cells from GSE1460. GSEA plots of signature from single-cell SOX4^+^ clusters of naive T cells enriched to thymocyte signature from microarray GSE1460. (G) UMAP plots of naive cells with averaged scaled expression of the top 100 genes from microarray of thymocyte signature (GSE1460). GSEA plots of thymocyte signature (GSE1460) enriched to single-cell naive T cells. (H) Volcano plot for comparison of CD31^+^ naive CD4^+^ T cells from healthy donors vs. CD31^+^ naive CD4^+^ T cells from thymectomy patients, source GSE72400 (left). Heatmap of normalized gene expression showing CD4^+^ SOX4^+^ signature (top 15 genes) in healthy and thymectomy individuals (middle). GSEA plot of single-cell CD4^+^ SOX4^+^ cluster signature enriched to signature of CD31^+^ naive CD4^+^ T cells from healthy donors (right).

When examining our published data on peripheral blood mononuclear cell (PBMC) aging (the ABF300 study),^7^ we noticed that naive T cells undergo significantly more pronounced age-dependent transcriptional remodeling compared with memory subsets (Figure 1A). To understand the remodeling of these cells at a deeper level, we focused on the analysis of naive cells only. Naive CD8^+^ T cells comprised 102,842 cells, and naive CD4^+^ T cells comprised 457,034 cells (STAR Methods). They clustered into three major clusters each (Figure 1B), and cluster 2, unlike other clusters, strongly declined with age (Figures 1C and S1A–S1C). Cluster 2 was marked by the expression of transcriptional factor *SOX4* (Figure 1D). Furthermore, other transcriptional regulators were specific for this cluster, such as *IKZF2*, *TOX*, and *TOX2* (Figure S1D). More broadly, gene signatures of SOX4^+^ subcluster were shared between naive CD8^+^ and CD4^+^ T cells (Figure 1E), suggesting commonalities in SOX4^+^ naive cells within both compartments. To further confirm these observations, we validated the existence and age-dependent behavior of SOX4^+^ subpopulation of naive T cells using publicly available data (syn22255433),^20^ which profiled PBMCs from 10 young (<35 years old) and 11 old (>64 years old) healthy donors (Figures S1E–S1G). The corresponding transcriptional signature of these subpopulations matched SOX4^+^ cells in the ABF300 data, as shown by pairwise gene set enrichment analysis (GSEA) (Figure S1H). Therefore, we conclude that SOX4^+^ naive cells represent a reproducible, transcriptionally distinct subpopulation of naive CD8^+^ and CD4^+^ T cells in humans.

### SOX4^+^ naive CD8^+^ and CD4^+^ T cells possess features of RTEs

The similarity of the transcriptional profiles of the CD8^+^ and CD4^+^ SOX4^+^ clusters suggested that there might be a common developmental or maturation process associated with these cells. SOX4 is reported to mark fetal and neonatal T cells.^21–23^ *TOX* and *TOX2* are associated with neonatal T cells and thymocyte development,^22,24,25^ even though they are also linked to T cell exhaustion.^26,27^ *IKZF2* is expressed during thymocyte development,^28^ besides its role in regulatory T cells.^29^ Therefore, we considered publicly available datasets describing human thymocytes and naive T cells to cross-reference the signature of the SOX4^+^ naive T cells. First, we compared our data with profiles of differentiation stages of thymocytes and T cells described in Lee et al.^30^ (GSE1460). The signature of total thymocytes was specifically enriched in the SOX4^+^ cluster and vice versa (Figures 1F and 1G). This suggested that SOX4^+^ naive T cells represent a transcriptionally distinct cluster of RTEs. This is consistent with the age-associated decline of SOX4^+^ cells that we observed (Figures 1C, S1B, and S1C), as it would be associated with an age-related decrease in thymic output.

Second, we analyzed transcriptional profiles of naive CD4^+^ T cells from neonatal thymectomy patients and healthy controls aged 1–5 years published by van den Broek et al.^17^ (GSE72400), dividing naive CD4^+^ T cells based on CD31 expression.^16,18^ We found that signatures of SOX4^+^ clusters were enriched specifically in the CD31^+^ naive CD4^+^ T cells from healthy controls and absent in naive cells from thymectomy patients (Figures 1H, S1I, and S1J; Table S1). Collectively, our analysis revealed that RTEs represent transcriptionally distinct subpopulation within naive T cells marked by specific expression of *SOX4* and other transcriptional regulators (*IKZF2*, *TOX*, and *TOX2*).

### SOX4^+^ naive T cells (RTEs) have distinct epigenetic landscapes

**Figure 2.**
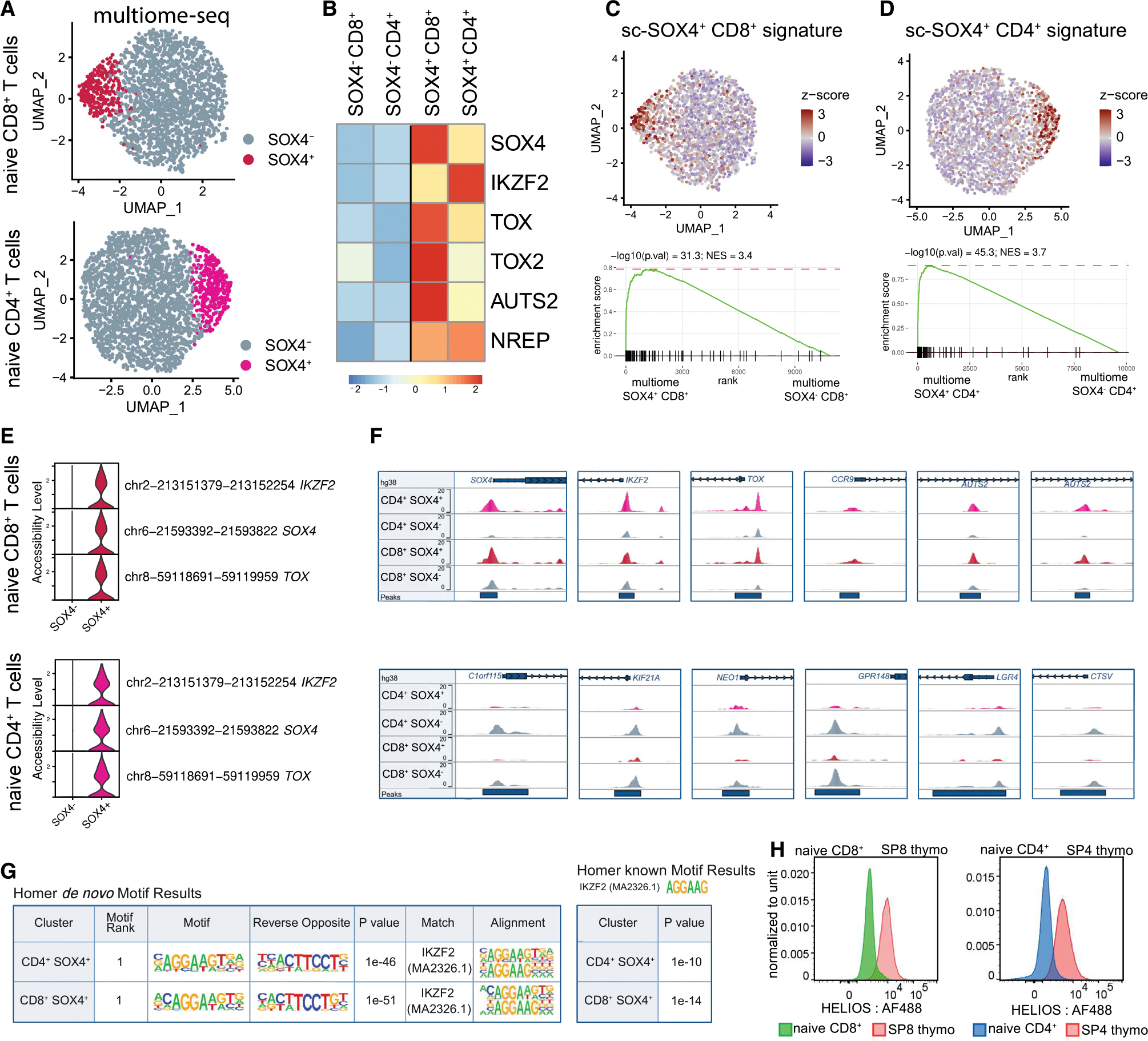
Single-nuclei analysis of chromatin accessibility of SOX4^+^ clusters (A) UMAP plots of T naive cells coming from multiome-seq, colored by cluster. (B) Heatmap of averaged normalized gene expression showing transcription factors of SOX4^+^ clusters. (C and D) UMAP plot of naive T cells with averaged scaled expression of sc-SOX4^+^ CD8^+^ (C) or CD4^+^ (D) gene signature. GSEA plots of sc-SOX4^+^ signature enriched to multiome SOX4^+^ signature. (E) Violin plots showing chromatin accessibility level for peaks shared between CD4^+^ and CD8^+^ SOX4^+^ clusters. (F) Coverage plots showing peaks of accessible chromatin near selected genes. (G) *De novo* and established TF motif analysis of SOX4^+^ cluster-specific regions. (H) Representative histograms showing HELIOS expression by naive T cells and SP thymocytes.

After defining RTEs based on their transcriptional state, we aimed to investigate the epigenetic profiles of these cells. Given the prevalence of SOX4^+^ cells in young (<35 years old) individuals, we performed single-nuclei multiome sequencing of sorted naive CD8^+^ and CD4^+^ T cells of young donors (Table S2). This assay enabled us to simultaneously detect gene expression and chromatin accessibility at single-cell resolution (STAR Methods). Clustering based on both transcriptional and epigenetic information confirmed the presence of two major populations inside naive T cells defined by the dichotomous expression and accessibility of the SOX4 gene (Figures 2A and 2B). The transcriptional portion of multiome profiles of SOX4^+^ clusters matched the signatures of the SOX4^+^ populations (RTEs) identified via single-cell RNA sequencing (scRNA-seq) data (Figures 2C and 2D). Moreover, the distribution of peaks showed not only distinct epigenomic landscapes of SOX4^−^ and SOX4^+^ clusters but also increasing DNA accessibility levels for key transcriptional factors *SOX4*, *IKZF2*, *TOX*, and other molecules associated with RTEs (Figures 2E and 2F). We further examined the enrichment of motifs within cluster-specific peaks. Both *de novo* motif analysis and the identification of established motifs showed the highest abundance of IKZF2 motifs in the SOX4^+^ cluster of both T cell compartments (Figure 2G; Tables S3 and S4). Consistently, high expression of HELIOS (*IKZF2*) is characteristic of human SP thymocytes, while only a fraction of circulating naive T cells expressed this protein (Figure 2H). Overall, our results revealed the epigenetically unique and specific state of SOX4^+^ naive T cells (RTEs), with *IKZF2* as their major transcriptional regulator.

### CD38 protein expression is a surface marker of the RTE T cells

**Figure 3.**
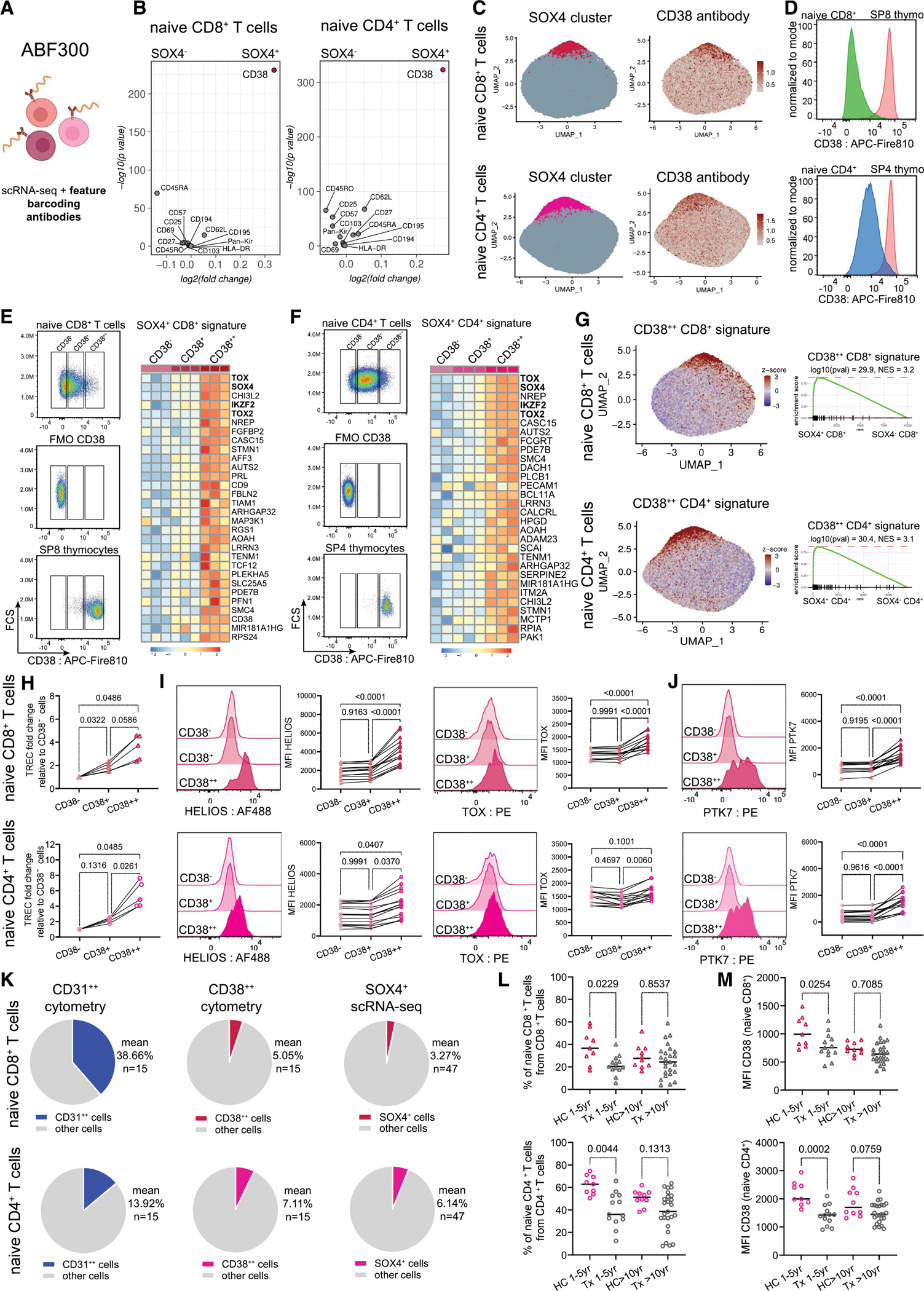
CD38 marks SOX4^+^ naive T cells (A) ABF300 schematic overview. (B) Volcano plots for comparison of normalized surface protein marker expression of SOX4^+^ vs. SOX4^−^ clusters of naive T cells. (C) UMAP plot of naive T cells showing SOX4^−^ and SOX4^+^ clusters (left) and normalized expression of CD38 antibody (right). (D) Representative histograms showing CD38 expression by naive T cells and SP thymocytes. (E and F) Representative dot plots of CD38 subset sorting strategy of naive CD8^+^ (E) and CD4^+^ (F) T cells (left). Heatmap of normalized gene expression showing SOX4^+^ signature (top 30 genes) in sorted CD38^−/+/++^ naive T cells (right). (G) UMAP plots with an averaged scaled expression of CD38^++^ signature (top 100 genes) from sorted CD38^++^ naive T cells to single-cell naive T cells. (H) Signal-joint (sj) TREC content of sorted CD38 subsets of naive T cells (<35 years old), normalized to CD38^−^ cell subset per each donor (*n* = 5). *p*.adj values by RM-ANOVA with Tukey’s multiple comparisons test using non-normalized data. (I) Flow cytometry plots of HELIOS or TOX co-expression with CD38 in naive T cells. Median fluorescence intensity (MFI) of HELIOS (*n* = 15) or TOX (*n* = 13) expression by CD38^−/+/++^ cells. (J) Representative flow cytometry plots of PTK7 and CD38 co-expression in naive T cells. MFI of PTK7 expression (*n* = 15) by CD38^−/+/++^ cells. (K) Pie charts showing the mean value of a percentage of CD8^+^ and CD4^+^ RTEs defined using different approaches. (L) Scatter plots showing the percentage of naive T cells of healthy controls and thymectomy patients (*n*_HC 1–5 years_ = 9; *n*_HC > 10 years_ = 10; *n*_Tx 1–5 years_ = 13; *n*_Tx > 10 years_ = 25). (M) Scatter plots showing MFI of CD38 expression of healthy controls and thymectomy patients (*n*_HC 1–5 years_ = 9; *n*_HC > 10 years_ = 10; *n*_Tx 1–5 years_ = 13; *n*_Tx > 10 years_ = 25). *p*.adj values by one-way ANOVA with Tukey’s multiple comparisons test.

Given the unique transcriptional and epigenetic landscape of SOX4^+^ naive cells (RTEs), we focused on identifying their protein surface marker, which would enable cell sorting and further characterization of these cells. The ABF300 scRNA-seq dataset^7^ also contained feature barcoding data to detect markers at the protein level (Figure 3A). Examining this data, we compared the surface protein expression of barcoded markers between SOX4^−^ and SOX4^+^ clusters (Figure 3B). We observed that elevated levels of expression of the CD38 protein were associated with SOX4^+^ clusters of both naive CD8^+^ and CD4^+^ T cells (Figures 3B, 3C, and S2A). CD38 was also highly expressed on the surface of human SP thymocytes, and a fraction of naive T cells possessed equally prominent levels of CD38 expression, suggesting CD38 high expression as a marker of RTEs (Figure 3D). To test the hypothesis that CD38 represents a marker of SOX4^+^ cluster (RTEs), we sorted CD38^−/+/++^ subsets of naïve T cells (Figure S2B; Table S2). To ensure an unbiased sorting strategy, we used the CD38 expression levels of SP thymocytes of a neonatal donor (2 weeks old) to establish gating for the CD38^++^ subsets (Figures 3E and 3F). CD38^−^ subsets were gated using CD38 fluorescence minus one (FMO) control (Figures 3E and 3F). After sorting, we performed bulk RNA-seq of CD38^−/+/++^ subsets. The data showed that the transcriptional signatures of sorted CD38^++^ naive T cells matched the corresponding signature of SOX4^+^ naive T cells derived from our single-cell data (Figures 3E–3G and S2C), suggesting that high CD38 expression is a valid marker for SOX4^+^ cells (RTEs). Additionally, we confirmed that CD38^++^ naive cells were enriched for TREC, indicating they egressed from the thymus more recently compared with other CD38 subsets (Figures 3H and S2D; Table S2). Next, we evaluated the CD38-based sorting strategy with respect to other markers of SOX4^+^ clusters. We evaluated the expression of HELIOS (*IKZF2*) and TOX (Table S2). We confirmed that only CD38^++^ naive CD8^+^ and CD4^+^ T cells express HELIOS and TOX proteins (Figures 3I, S2E, and S2F). Altogether, these data confirmed that RTEs (defined as a transcriptional population of SOX4^+^ naive T cells; Figures 1 and 2) could be identified by high surface expression of the CD38 protein.

We further evaluated previously described markers of CD4^+^ RTEs.^18,19,31^ Firstly, we considered PTK7, a marker of the “most immature” CD4^+^ RTEs,^17^ which was expressed by thymocytes with overlapping expression on naive cells (Figure S2G). We found that the PTK7-expressing cells were present among the CD38^++^ naive cells in both T cell compartments (Figures 3J and S2H). Secondly, we examined the expression of CD31 and observed a correlation in expression with the CD38 molecule (Figure S2I). Further, we wanted to compare CD38-based gating and CD31-based gating. We employed a similar gating approach for CD31^−/+/++^ cell subsets as for CD38 subsets (Figure S2J). We observed that PTK7 and HELIOS expressions were also generally associated with CD31 expression (Figure S2K). However, the enrichment of the corresponding signal was significantly lower compared with CD38-based gating (Figure S2L), suggesting that CD38 is a specific surface marker of the RTEs. Furthermore, we observed a significant difference in RTE proportions defined by CD38 or CD31 within naive cells. We showed by flow cytometry that the proportion of CD38^++^ subpopulation of naive cells was ~5% of naive T cells in young (<35 years old) individuals (Figures 3K and S2M), which matched the fractions of SOX4^+^ cells from scRNA-seq data (Figures 3K and S1B). On the other hand, the CD31^++^ population was ~40% of naive CD8^+^ T cells and ~15% of naive CD4^+^ T cells, which is considerably larger than the corresponding fraction of RTEs defined by scRNA-seq (Figure 3K).

Lastly, we considered if CD38 expression on naive T cells changes in patients who underwent thymectomy. To this end, we analyzed previously unpublished data generated as a part of studies by van den Broek et al.^17,32^ The cohort included healthy controls and neonatal thymectomy patients.^17^ Data showed a decline in the proportion of naive cells in thymectomy patients aged 1–5 years (Figures 3L and S2N). Furthermore, CD38 expression within naive T cells also declined in thymectomy patients aged 1–5 years, confirming that high CD38 expression serves as a marker of RTEs (Figure 3M). There was no statistical difference between healthy and thymectomy donors older than 10 years (Figures 3L and 3M), which was expected since these surgeries are heart-focused and often leave some thymic tissue that can regrow over time.^33,34^ This observation further strengthens the conclusion that CD38 is a robust universal marker for the precise identification of both CD8^+^ and CD4^+^ RTEs.

### Immunophenotypic characterization of RTE naive T cells

**Figure 4.**
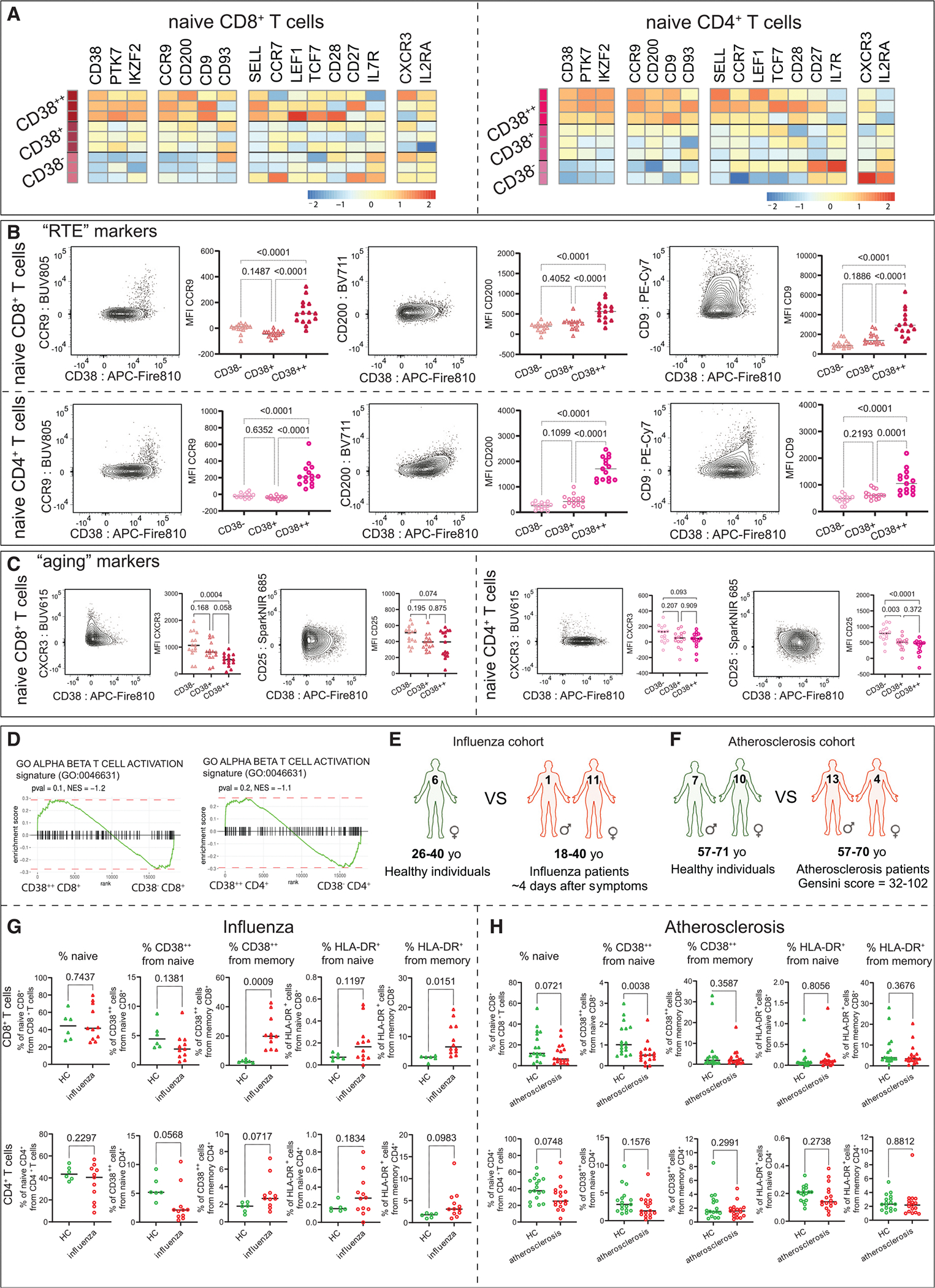
Phenotype analysis of CD38^++^ RTEs and inflammatory conditions (A) Heatmap of normalized gene expression of selected genes in sorted CD38^−/+/++^ naive T cells. (B) Representative flow cytometry plots and MFI of CCR9, CD200, and CD9 expression in CD38^−/+/++^ naive T cells. (C) Representative flow cytometry plots and MFI of CXCR3 and CD25 expression in CD38^−/+/++^ naive T cells. *p*.adj values by one-way ANOVA with Tukey’s multiple comparisons test (*n* = 15). (D) GSEA plots of GO CD8/CD4 alpha/beta T cell activation signature (GO: 0046631) to CD38^++^ vs. CD38^−^ naive CD8^+^/CD4^+^ T cells. (E) Scheme of influenza cohort. (F) Scheme of atherosclerosis cohort. (G) Scatter plots showing the percentage of T cell subsets between HC and influenza patients. *p* values by unpaired t test (*n*_HC_ = 6; *n*_infl_ = 11). (H) Scatter plots showing the percentage of T cell subsets between HC and atherosclerosis patients. *p* values by unpaired t test (*n* = 34).

Given the ability to distinguish RTEs via CD38 expression, we immunophenotyped these cells at a deeper level using samples from 15 young (<35 years old) individuals (Table S2) and custom spectral flow cytometry panels (Table S5). Based on our transcriptional data (Figures 3E and 3F), we selected *CCR9*, *CD200*, and *CD9* as additional potential surface markers of this population (Figure 4A). Indeed, these proteins were expressed at the highest intensities by CD38^++^ subsets (Figure 4B). The identification of additional markers of RTEs is beneficial for practical applications such as RTE gating. The gating of RTEs described above (Figure 3) involved thresholding on the CD38^++^ population by comparison with thymocytes. We found that CCR9 expression provides the threshold of CD38 expression that closely matched the one derived from the thymocyte data (Figures S3A and S3B). Therefore, including CCR9 in the potential RTE panel design can be beneficial when human thymocytes are not available as a positive gating control.

Of note, within the adult thymuses, both CD38^+^ and CD38^−^ SP populations were present (Figure S4A). CD38^+^ SP thymocytes co-expressed the additional RTE markers (PTK7, CCR9, CD200), while CD38^−^ cells did not (Figure S4B). To investigate the CD38^−^ cells further, we considered a publicly available dataset containing scRNA-seq data of pediatric (3 months–13 years old) and adult (24–35 years old) thymic cells, which revealed specific clusters of CD38^+^ and CD38^−^ SP cells consistently with flow cytometry data (Figure S4C). The CD38^−^ populations increased with age and lacked the SOX4 signature (Figures S4D and S4E). Furthermore, a subset of CD38^−^ SP cells expressed FAS, CD69, CD103, CXCR3, and PD1 and lacked CD45RA and CCR7, suggesting that these could be mature recirculating or tissue-resident T cells^35–39^ (Figure S4F).

Further, we evaluated the expression of the classical naive T cell markers^40–42^ by CD38 subsets. CD62L and CCR7 expression were higher in the CD38^++^ population of naive CD8^+^ T cells, while only CD62L was associated with CD38 levels in naive CD4^+^ T cells (Figure S5A). CD127 demonstrated the opposite pattern of increased expression in CD38^−^ naive CD4^+^ T cells (Figure S5A). CD38^++^ CD4^+^ RTEs generally increased the expression of CD28 without any change in naive CD8^+^ T cells (Figure S5A). CD45RA and CD27 expression did not have a clear CD38-associated pattern (Figure S5A). Lastly, two major naive transcription factors, LEF1 and TCF1 kept high expression levels independent of CD38 expression (Figure S5A). This can suggest that the basal naive T cell characteristics are common between RTEs and mature naive T cells.

Lastly, previous studies^7,43–45^ reported that CD25 and CXCR3 are surface proteins associated with naive T cell aging. Specifically, naive CD8^+^ T cells are found to gain expression of CXCR3 with age,^43,45^ and naive CD4^+^ T cells elevate expression of CD25.^7,44^ Therefore, we immunophenotyped naive T cells using CD25 and CXCR3, among other proteins. Our data demonstrated an inverse relation of these markers to CD38 (Figure 4C). CD38^−^ naive CD8^+^ T cells increased CXCR3 expression, while alternation in CD25 expression did not reach statistical significance. Conversely, CD38^−^ naive CD4^+^ T cells expressed enhanced levels of CD25, but the CXCR3 expression did not change significantly (Figure 4C).

### CD38 expression and RTEs in the context of inflammation

CD38 is known as a T cell activation marker.^46,47^ Therefore, we considered whether high levels of CD38 on RTEs were related to their activation status. Transcriptionally, we found that the CD38^++^ naive signature was not enriched in T cell activation pathways (Figure 4D). To validate this, we investigated CD38 expression by T cells in both *in vivo* and *in vitro* contexts. Firstly, we examined the impact of acute and chronic inflammatory conditions using cohorts of influenza patients (acute inflammation) and atherosclerosis patients (chronic inflammation) (Figures 4E and 4F; Table S2). In the context of acute inflammation, the proportion of CD38^++^ and HLA-DR^+^ memory CD8^+^ T cells was significantly elevated, indicating an acute inflammatory response consistent with previous observations^47^ (Figure 4G). However, the proportion of naive cells and CD38^++^ naive cells did not increase under this condition, indicating that acute influenza did not impact thymic output (Figure 4G). In atherosclerosis patients, there was a decline in CD38^++^ naive CD8^+^ T cells, while HLA-DR^+^ and CD38^++^ memory T cells were not different in this condition (Figure 4H). This supports the hypothesis that low-density lipoprotein (LDL) may accelerate thymic involution and potentially contribute to atherosclerosis progression.^48^ Additionally, naive T cells express low to negligible levels of activation molecule HLA-DR in contrast to memory T cells (Figures 4G and 4H).

Secondly, in the *in vitro* model, we utilized homeostatic proliferation of CD38-expressing RTEs. We sorted CD8^+^ and CD4^+^ RTEs, but due to the limited number of naive CD8^+^ T cells, we used a more relaxed threshold and sorted CD38^+^ naive CD8^+^ T cells (juvenile cells—a broader population enriched for RTEs) (Figure S5B). We cultured CD38^+/hi^ naive cells under homeostatic conditions (Figure S5B; Table S2), and CD8^+^ and CD4^+^ RTEs exhibited minimal expression of activation markers (HLA-DR, CD69) (Figure S5C). Focusing on HLA-DR^−^CD69^−^ cells (Figure S5D), we found that CD8^+^ RTE cells underwent six proliferation cycles during the 10-day incubation, whereas CD4^+^ RTE cells from only four out of seven donors reached five proliferation cycles (Figure S5E). CD38^+/hi^ RTEs demonstrated increased CD38 expression during intensive homeostatic proliferation within the first 1–3 proliferative cycles compared with steady-state conditions. However, CD38 expression began to decrease from the 3–4 proliferation cycles (Figure S5F). This decline was accompanied by the emergence of CD38^−/lo^ cells (Figure S5G). Our data also demonstrated that CCR9 and PTK7 were completely lost within 1–3 proliferation cycles (Figure S5H). These findings suggested that RTEs ceased expression of CD38 and other RTE markers and transited into mature naive T cells during prolonged homeostatic proliferation.

### Age-dependent compositional remodeling of naive T cells

**Figure 5.**
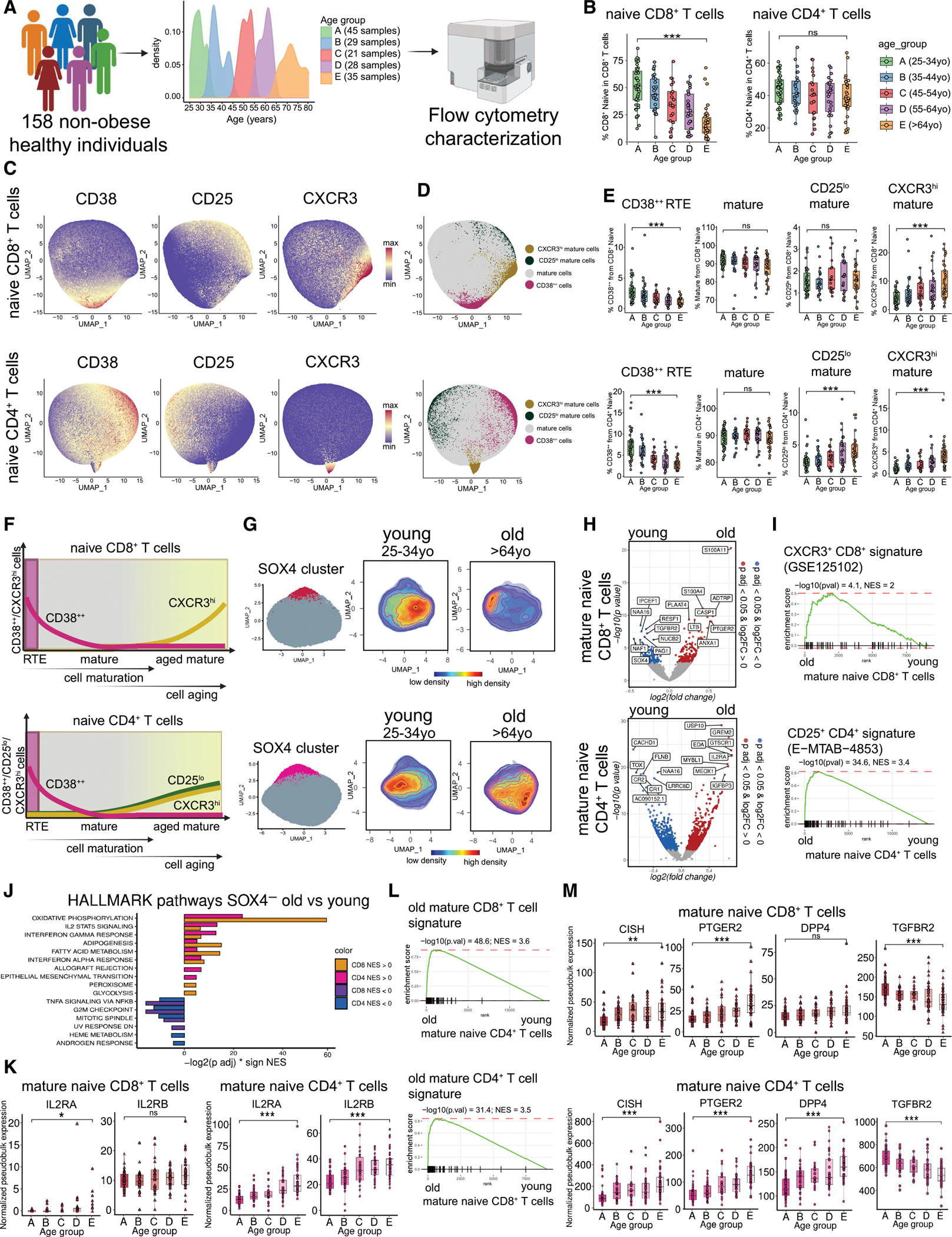
Age-dependent remodeling of naive T cells (A) Schematic overview of aging cohort cytometry processing. (B) Boxplots showing the percentage of naive T cells using flow cytometry by the age groups A–E, *p*.adj by post hoc Dunn’s test after one-way Kruskal-Wallis test with Holm correction method (*n* = 158 overall [A = 45, B = 29, C = 21, D = 28, and E = 35]). *p.*adj values were additionally corrected with Bonferroni method by the number of subpopulations in the comparison. (C) UMAP plots with a surface expression of CD38, CD25, and CXCR3 in flow cytometry data. (D) UMAP plots showing subpopulations of naive T cells specified by threshold gating. (E) Boxplots showing the percentage of naive T cell subpopulations using flow cytometry by the age groups A–E, *p.*adj same as in (B). (F) Scheme of maturation and aging of naive T cells. (G) UMAP density plots characterizing the distribution of SOX4^+^ and SOX4^−^ naive T cells between young and old individuals. (H) Volcano plot for comparison of old vs. young SOX4^−^ clusters in naive cells. (I) GSEA plot of CXCR3^+^ naive CD8^+^ T cell signature (GSE125102) enriched to single-cell old naive CD8^+^ T cells (top). GSEA plot of CD25^+^ naive CD4^+^ T cell signature (E-MTAB-4853) enriched to single-cell old naive CD4^+^ T cells (bottom). (J) Significantly (*p*.adj < 0.05) enriched pathways for comparison of old vs. young SOX4^−^ naive CD8^+^ and CD4^+^ T cells. (K) Boxplots showing normalized pseudobulk expression of IL-2RA and IL-2RB in SOX4^−^ clusters of naive T cells, *p.*adj using Wald test from DESeq2 (CD4^+^, *n* = 166; CD8^+^, *n* = 164). (L) GSEA plot of old SOX4^−^ naive CD8^+^ T cell signature enriched to SOX4^−^ naive CD4^+^ T cells and vice versa. (M) Boxplots showing normalized pseudobulk expression of selected genes in SOX4^−^ clusters of naive cells, *p.*adj using Wald test from DESeq2 (CD4^+^ *n* = 166; CD8^+^ *n* = 164). *p.adj < 0.05, **p.adj < 0.01, ***p.adj < 0.001, ns, not significant.

To deeply understand the age-associated remodeling of naive cells, we profiled naive T cells from 158 healthy individuals across different ages using a cytometry panel of 35 markers (Figure 5A; Tables S2 and S5). For analysis, samples were split into 5 age groups with 10-year-long intervals (25–34, 35–44, 45–54, 55–64, and >64 years old), resulting in 21–45 individuals per age group (Figure 5A). After quality control filtering, when we excluded doublets, debris, and dead cells from our dataset, we clustered PBMCs to identify naive cells (STAR Methods). Naive CD8^+^ T cells were defined as CD3^+^CD8^+^CD4^−^γδTCR^−^ TCRVα7.2^−^CCR7^+^CD45RA^+^FAS^−^ cells (Figure S6A), and naive CD4^+^ T cells were defined as CD3^+^CD8^−^CD4^+^γδTCR^−^CD127^+^ CD25^lo^CD45RA^+^FAS^−^ cells (Figure S6B). Consistent with prior observations,^7,20,49^ the proportion of naive CD8^+^ T cells significantly decreased with age, while the proportion of naive CD4^+^ T cells remained unchanged during aging (Figure 5B). Next, we examined the age-dependent behavior of distinct naive T cell subpopulations identified by expression of CD38, CXCR3, or CD25 (Figures 5C, 5D, and S6C). The dominant subset among naive T cells consisted of mature naive T cells lacking expression of either CD38, CXCR3, or CD25. Consistent with the expectations, the results showed that CD38^++^ cells were continuously declining with age (Figures 5E and S6D), and PTK7-expressing cells represented their subset (Figure S6E). We also observed an accumulation of CD25^lo^ naive CD4^+^ T cells with age (Figures 5E and S6D), consistent with previous reports^7,44^ (Figures 5E and S6D). Additionally, we observed that distinct cell subsets marked by CXCR3 high expression increased within both naive CD8^+^ and CD4^+^ T cells (Figures 5E and S6D). In naive CD8^+^ T cells, this subpopulation has been previously described as T memory cells with naive phenotype (T_MNP_ cells).^43,45^ However, to the best of our knowledge, age-associated increase of CXCR3^hi^ naive CD4^+^ T cells has not been reported to date. These observations demonstrated that there are three axes of naive T cell aging—(1) the CD4^+^ T cell-specific axis associated with CD25^lo^ cell accumulation, (2) the CD8^+^ and CD4^+^ shared axes associated with accumulation of CXCR3^hi^ cells, and (3) decline of CD38^++^ RTEs (Figure 5F). Neither CD8^+^ nor CD4^+^ CXCR3^hi^ cells would be classified as memory-like, given their high CD45RA expression and low FAS expression (Figure S6F). This indicated that the presence of the CXCR3^hi^ population may be driven by the maturation/aging process within the *bona fide* naive T cells.

### Age-dependent transcriptional remodeling of mature non-RTE naive T cells

Given the apparent age-dependent remodeling of mature, non-RTE, naive T cells, we examined the transcriptional aging signatures of mature naive T cells (Figure 5G). To that end, we analyzed transcriptional profiles of SOX4^−^ mature naive cells from old (>64 years old) and young (<35 years old) donors using data from ABF300 cohort.^7^ Indeed, these cells demonstrated significant remodeling associated with age (Figure 5H; Table S6). Differential expression associated with the aging of mature naive CD8^+^ T cells was strongly enriched in signatures of CXCR3^+^ naive CD8^+^ T cells derived from published data (GSE125102)^43^ (Figure 5I). Similarly, a comparison of the aging signatures of naive CD4^+^ T cells against published signatures of sorted CD25^+^ naive CD4^+^ T cells (E-MTAB-4853)^44^ demonstrated significant enrichment (Figure 5I).

The changes in multiple pathways were shared between mature naive CD8^+^ and CD4^+^ T cells (Figure 5J), including an increase in the oxidative phosphorylation pathway or a decrease in the G2M checkpoint and the mitotic spindle pathways (Figure 5J). The interleukin (IL)-2-STAT5 pathway was enriched in both naive CD8^+^ and CD4^+^ T cells with age, but the signal was more pronounced in aged naive CD4^+^ T cells. Furthermore, IL-2RA and IL-2RB expression was increased only in naive CD4^+^ cells (Figure 5K), consistent with the cytometry observation.^7,44,50^ The substantial degree of shared aging pathways suggested the existence of the universal aging program of naive T cells. Indeed, aging signatures of mature naive CD8^+^ and CD4^+^ T cells showed significant mutual enrichment (Figure 5L). Further examination of this program revealed that many genes previously associated with overall T cell aging were an intrinsic part of the aging of mature naive T cells. For instance, transcriptional regulator *CISH* increased with age in mature naive cells from healthy individuals (Figure 5M) and is described to be involved in elevated age-dependent mitochondrial release.^50^ Secondly, the prostaglandin E2 (PGE2) receptor (*PTGER2*) increased in mature naive cells with age (Figure 5M). PGE2 stimulation is suggested to be associated with features of replicative senescence in human CD8^+^ T cells.^51^ Next, we determined elevated *DPP4* gene expression in mature naive CD4^+^ T cells (Figure 5M), which is associated with memory-like phenotypes.^52,53^ Lastly, transforming growth factor β (TGF-β) receptor (*TGFBR2*) expression declined in mature naive T cells (Figure 5M), which plays a role in the activation capabilities of these cells.^54^ Taken together, naive CD8^+^ and CD4^+^ T cells age according to similar transcriptional patterns, which might influence their functional capability during the immune response.

### The age-dependent functional differences in naive T cells

**Figure 6.**
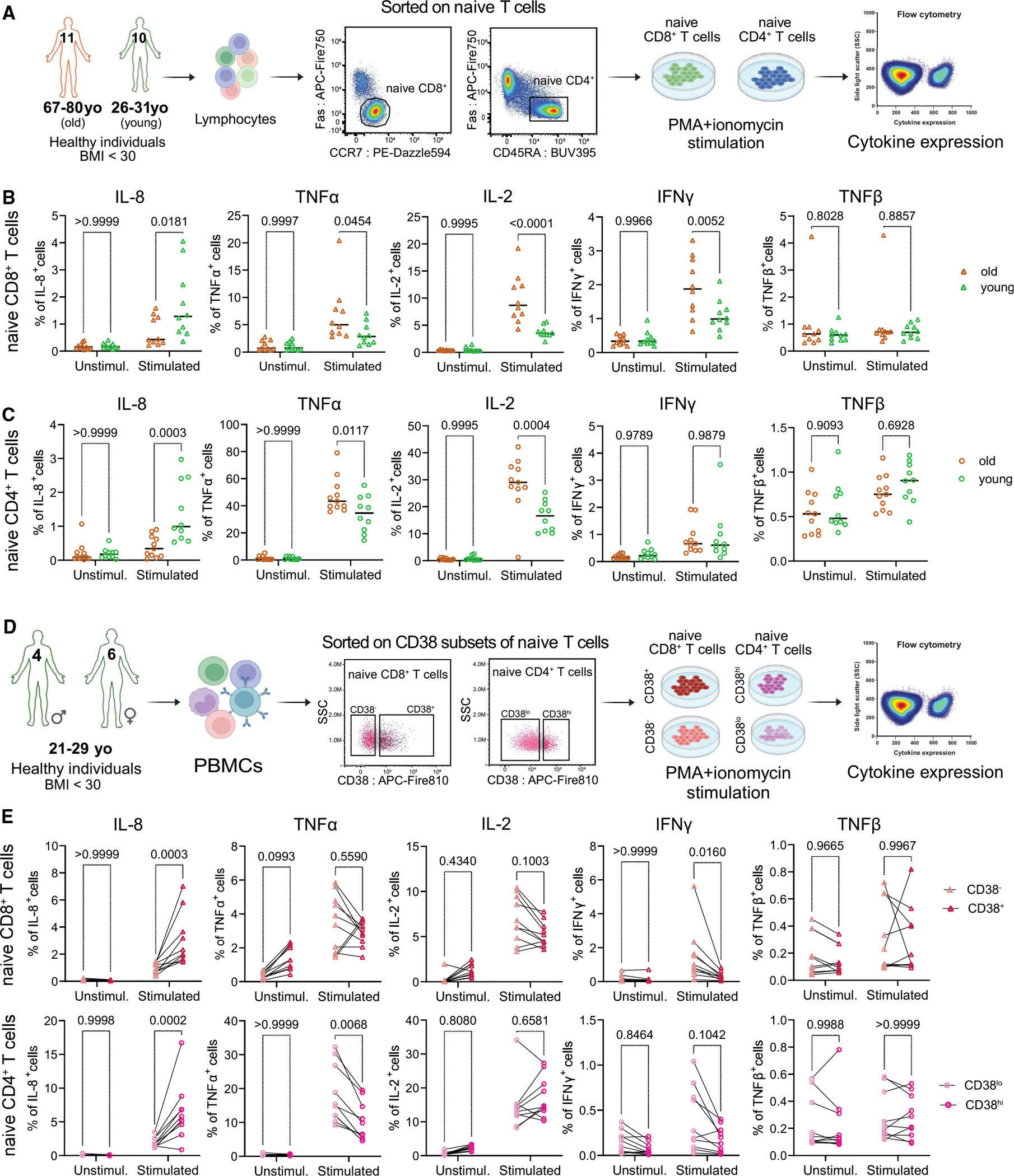
Age-dependent functional remodeling of naive T cells (A) Scheme of evaluation of age-dependent functional change of naive T cells. (B and C) Scatter plots showing the percentage of cytokine-positive naive CD8^+^ (B) and CD4^+^ (C) T cells in unstimulated and PMA/ionomycin-stimulated conditions for young and old age groups. (D) Scheme of evaluation of functional change of CD38 subsets of naive T cells. (E) Scatter plots showing the percentage of cytokine-positive cells in CD38 subsets of naive T cells in unstimulated and PMA/ionomycin-stimulated conditions. *p.*adj values by two-way ANOVA with Tukey post hoc (*n* = 10).

Consequently, we examined the functional changes between naive cells of 10 young (<35 years old) and 11 old (>64 years old) donors (Table S2). To that end, we sorted naive CD8^+^ and CD4^+^ T cells, stimulated them with phorbol 12-myristate 13-acetate (PMA) and ionomycin, and evaluated their capability to express primary inflammatory cytokines (Figure 6A). We observed a significant difference in stimulation response between young and old cells. First, old naive cells expressed significantly less IL-8 (Figures 6B and S7A). Second, naive CD8^+^ T cells from older individuals increased expression of tumor necrosis factor alpha (TNF-α), IL-2, and interferon gamma (IFN-γ), while expression of TNF-β did not differ (Figures 6B and S7A). Regarding the CD4^+^ T cell compartment, naive cells from older individuals demonstrated increased expression of TNF-α and IL-2, while expression of IFN-γ and TNF-β did not vary between age groups (Figures 6C and S7A). Therefore, our data demonstrated that naive T cells from old and young individuals also differ in their functional aspects.

Identification of CD38 as a marker of RTEs allowed us to understand the contribution of RTE cells toward age-related functional changes in naive T cells. Thus, we sorted CD38 subpopulations of naive T cells of 10 young (<35 years old; Table S2) donors, stimulated them with PMA/ionomycin, and measured their cytokine expression (Figure 6D). Indeed, the short-time activation program of RTEs/juvenile cells vs. mature cells differed significantly. First, RTEs/juvenile cells significantly increased expression of IL-8 (Figures 6E and S7B), consistent with previous reports.^17,55–57^ Second, mature naive CD4^+^ but not CD8^+^ T cells elevated expression of TNF-α (Figures 6E and S7B). However, only mature naive CD8^+^ T cells had elevated IFN-γ expression upon stimulation (Figures 6E and S7B). The expression of IL-2 and TNF-β was comparable between RTEs and mature naive cells in both compartments (Figures 6E and S7B). In summary, our results showed age-related changes in the functionality of naive T cells, highlighting that some of these alterations can be associated with functional distinctions between RTEs and mature naive T cells.

### Transcriptional activation programs of RTE and mature subsets of naive T cells

**Figure 7.**
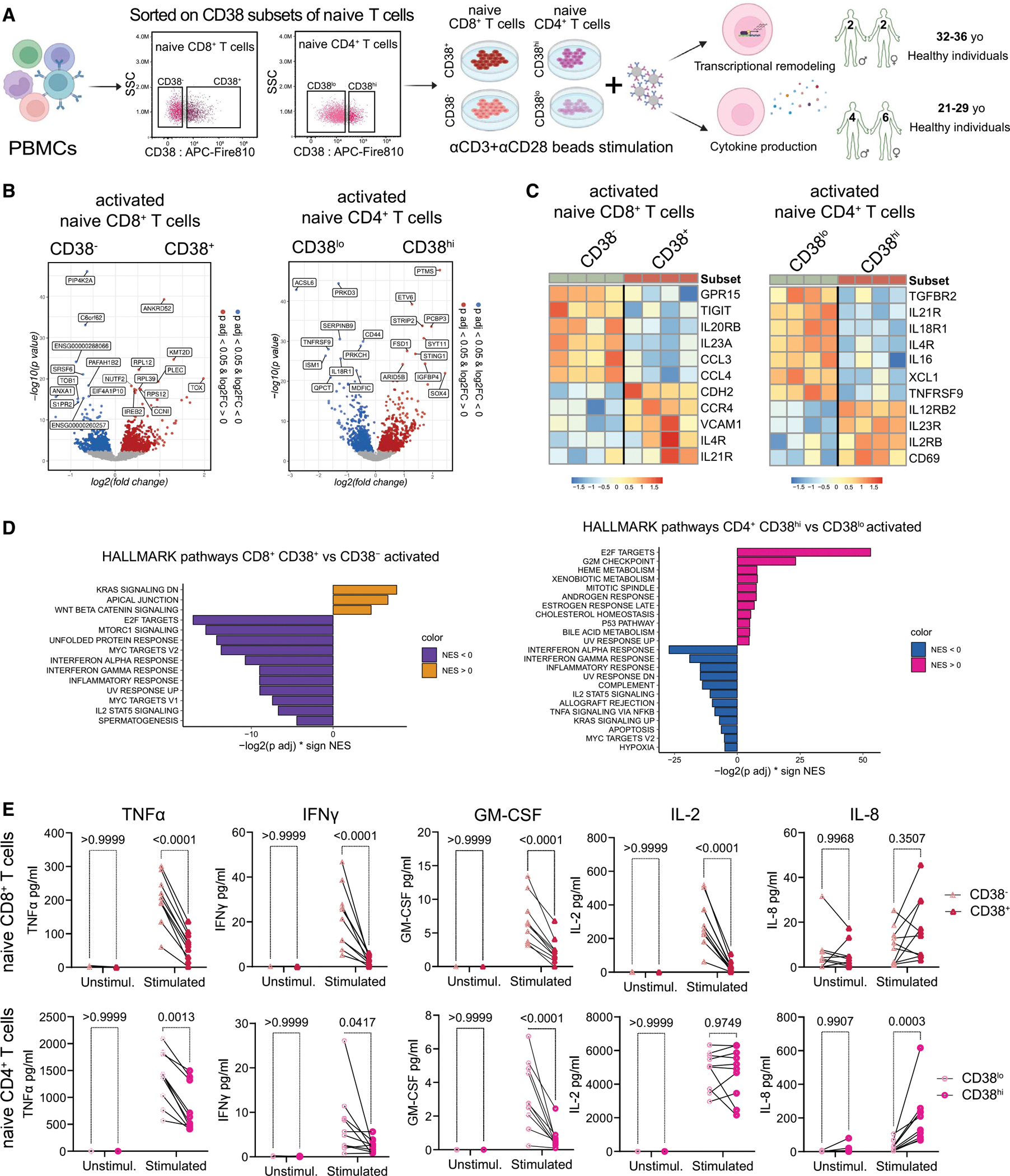
Activation programs of RTEs and mature naive T cells (A) Overview illustrating the analysis of activation programs of naive T subsets. (B) Volcano plot for comparison of CD38^+/hi^ vs. CD38^−/lo^ activated naive cells. (C) Heatmaps of normalized gene expression showing selected genes in CD38^−/lo^ and CD38^+/hi^ naive T cells. (D) Significantly (*p*.adj < 0.05) enriched pathways for comparison of CD38^+/hi^ vs. CD38^−/lo^ activated naive T cells. (E) Scatter plots showing cytokine production by CD38 subsets of naive cells in unstimulated and beads-activated conditions (*n* = 10), *p.*adj values by two-way ANOVA with Tukey post hoc.

To better understand the difference in functionality, we performed transcriptional profiling and analyzed actual cytokine secretion upon activation of CD38^−/lo^ and CD38^+/hi^ cells. To that end, we sorted CD38 subpopulations of naive T cells of young (<35 years old) donors (Table S2), stimulated them with aCD3/aCD28 beads for 24 h, and performed bulk RNA-seq and determined cytokine levels (Figure 7A). We observed that transcriptional reprogramming of stimulated RTEs/juvenile cells vs. mature cells differed significantly in both T cell compartments (Figure 7B), as evidenced by the increase of 844 (CD8)/742 (CD4) genes and the decrease in 819 (CD8)/648 (CD4) genes (Table S7) in CD38-expressing RTE cells compared with mature cells. A number of differentially expressed genes were associated with proinflammatory actors such as *CCL4*, *CCL3*, *IL4R*, and *IL21R* for naive CD8^+^ T cells (Figure 7C), and *XCL1*, *IL21R*, *IL23R*, or *IL12RB2* for naive CD4^+^ T cells (Figure 7C).

Pathway enrichment analysis showed that inflammatory signaling pathways were elevated in the activated mature cells (Figure 7D). Therefore, we analyzed the actual cytokine production by naive T subsets (Figure 7E). Even though cytokine production by naive cells is generally low (Figure S7C), we indeed observed strongly enhanced production of inflammatory cytokines, such as TNF-α, IFN-γ, and GM-CSF, in mature naive cells (Figure 7E). Furthermore, mature naive CD8^+^ T cells elevated IL-2 production upon TCR trigger (Figure 7E). Reversely, CD4^+^ RTEs produced more IL-8 compared with mature naive CD4^+^ T cells, but without significant change in the CD8^+^ T cell compartment (Figure 7E). Taken together, these data indicate these subsets undergo different transcriptional reprogramming during activation and have distinct capability to secrete cytokines.

## DISCUSSION

Using large-scale single-cell resolution profiling of healthy human PBMCs,^7^ we analyzed naive CD8^+^ and CD4^+^ T cells in depth and identified a transcriptionally and epigenetically distinct *SOX4*^+^*IKZF2*^+^*TOX*^+^ subset of naive CD8^+^ and CD4^+^ T cells that progressively decreased with age. Further analysis determined that this subpopulation can be classified as RTE cells. In fact, the importance of the key transcription factors of the RTEs is described in the general context of age-dependent remodeling.^21,22,58^ Specifically, a decrease in *IKZF2* expression is associated with age-dependent remodeling of naive CD4^+^ T cells.^58^ Moreover, adult naive CD8^+^ T cells are shown to have decreased levels of expression of *SOX4* and *TOX* compared with newborn CD8^+^ T cells.^21,22^ The unprecedented depth and age range of the ABF300^7^ dataset allowed us to place these previous observations into the context of compositional remodeling of naive T cells and establish the distinct subset of CD8^+^ and CD4^+^ RTEs.

Leveraging this definition, we identified the CD38 molecule as a surface marker for the CD8^+^ and CD4^+^ RTE cells. CD38 expression is suggested to play a role in human thymocyte development,^59^ and SP thymocytes share a high expression level of CD38 with RTEs, which can guide their accurate identification in the naive T cell pool using flow cytometry. In fact, in 1998,^60^ the shared intensity of CD38 expression between thymocytes and neonatal naive CD4^+^ T cells implies that this cell population represents an immature transitional population between thymocytes and the more mature naive CD4^+^ T cells in adults. CD38 provides not only a marker of RTEs for the naive CD8^+^ T cells but also a universal marker of RTEs applicable to both T cell compartments.

To further dissect the age-related behavior of naive T cells, we profiled a cohort of 158 individuals ranging in age from 25 to 85 years using a custom spectral cytometry panel focusing specifically on these proteins. The proportions of the CD38^++^ RTEs declined with age within both naive CD8^+^ and CD4^+^ T cells, reflecting decreasing thymic function.^61^ This contrasts with the observed increase in CD38 expression in peripheral tissues, where it is associated with aging.^62,63^ Related to aging, we observed an accumulation of CD25^lo^ naive CD4^+^ T cells with age, consistent with previous observations.^7,44^ We also found that CXCR3^hi^ naive T cells increased with age within CD8^+^ and CD4^+^ naive cells. These cells were distinct from CD25^lo^ or CD38^++^ cells and have been previously described as T_MNP_ cells.^45^ Age-associated accumulation of this subset in naive CD4^+^ T cells has not been reported to date.

The reliable RTE marker is an important readout of the efficacy of potential immune “rejuvenation” intervention as a direct measure of reversal of thymic involution, such as reported in calorie restriction and physical exercise studies.^64,65^ Several studies^48,66–68^ have suggested that infections and comorbidities (such as obesity, LDL levels, etc.) can impact the thymus, causing accelerated thymic involution and disrupting peripheral immune balance by altering RTE production. Thus, RTE quantification could provide critical insights into the degree of immune disruption and thymic involution in these conditions, aiding in the assessment of disease progression.

In summary, our study robustly established CD8^+^ and CD4^+^ RTEs as CD38^++^*SOX4*^+^ cells, which allowed us to decouple compositional and intrinsic remodeling of naive T cell pool during aging in humans. Our observations further suggested the existence of three aging axes of naive T cells—(1) decrease in CD38^++^ naive cells (RTEs), (2) increase in CXCR3^hi^ naive cells, and (3) CD4-specific increase in CD25^lo^ naive cells.

### Limitations of the study

Previous studies^69,70^ have demonstrated that CD38 expression is sensitive to the duration of cryopreservation, with longer storage periods resulting in a lower proportion of CD38^+^ cells. Therefore, it is crucial to account for the cryopreservation duration when evaluating CD38 expression levels. Comparisons of CD38 expression between samples with significantly different cryopreservation periods may yield misleading results. Furthermore, due to the evolving definitions of memory-like naive T cells and memory T cells exhibiting naive features,^45,52,71^ accurately delineating *bona fide* naive T cells using flow cytometry remains challenging.

## RESOURCE AVAILABILITY

### Lead contact

Further information and requests for resources and reagents should be addressed to the lead contact, Maxim N. Artyomov (martyomov@wustl.edu).

### Materials availability

This study did not generate new, unique reagents. For further information and material requests, please contact Maxim N. Artyomov (martyomov@wustl.edu).

### Data and code availability

- Raw and processed RNA-seq and scRNA-seq/multiome-seq data generated in this study and spectral cytometry data have been deposited at Synapse repository and are publicly available as of the date of publication. Links for the single-cell online browser and interactive heatmaps have been deposited at the Synapse repository. The accession number is listed in the key resources table.
- The pipeline with code to process and visualize spectral cytometry data is available in a GitHub repository and is publicly available as of the date of publication. DOI is listed in the key resources table.
- The lead contact can provide any additional information required to reanalyze the data reported in this paper upon request.

## STAR★METHODS

### EXPERIMENTAL MODEL AND STUDY PARTICIPANT DETAILS

#### Blood samples used in the study

1. Aging cohort: The samples for cross-section aging profiling in Figure 5 were taken from a sample collection collected under the original ABF300 study.^7,49^
2. Influenza cohort: The EDFLU study was approved by the Washington University in St. Louis Institutional Review Board (approval number 2017–10-220). Potential subjects with a positive clinical influenza real-time reverse-transcription polymerase chain reaction test and ongoing influenza-like illness symptoms within the past 24 hours were approached for potential enrollment. Written informed consent was obtained from each subject or their legally authorized representative.
3. Atherosclerosis cohort: Subjects with suspected coronary artery disease from the Coronary Assessment in Virginia cohort (CAVA) were recruited for the study through the Cardiac Catheterization Laboratory at the University of Virginia Health System, Charlottesville, VA, USA. All participants provided written informed consent before enrollment, and the study was approved by the Human Institutional Review Board (IRB approval number 15328). Peripheral blood was obtained from these participants prior to catheterization.
4. Thymectomy cohort: Thymectomy patient data was generated as a part of studies by van den Broek et al.^17,32^ and previously unpublished. Shortly, patients who had undergone thymectomy within the first month of life because of surgery to treat congenital heart defects at the Wilhelmina Children’s Hospital were included. Blood samples were taken at between 1 to 5 years and after 10 years following neonatal thymectomy. For more information, please see the original studies.^17,32^

#### Samples collected as a part of the current study

##### Blood

1. Healthy validation samples-1: For validation and functional assay purposes, additional blood samples from healthy participants were collected in the same way as samples from the ABF300 study^7,49^ (IRB approval number 201804084). Shortly, all participants were recruited from the St. Louis area and provided written informed consent. Caucasian, non-obese (BMI < 30) males and females were enrolled. Participants were given a screening questionnaire to establish their health status. Only non-smokers without a history of cancer, chronic inflammatory conditions (arthritis, Crohn’s disease, colitis, dermatitis, fibromyalgia, or lupus), or blood-borne infections (HIV, hepatitis B, and C) were included. Participants who reported flu symptoms or cold in the previous month were excluded. Venous blood (~100 ml) was collected into sodium-heparin BD vacutainer tubes in the morning (7–10 AM) after an overnight fast.
2. Healthy validation samples-2: For the transcriptional profiling of activated CD38 subsets of naive CD8^+^ and CD4^+^ T cells (Figure 7), TREC content (Figures 3H and S2D), and homeostatic proliferation (Figure S5), PBMCs were obtained from the leukoreduction filters after platelet apheresis of anonymous healthy blood donors of both sexes.

##### Thymuses

1. *Pediatric thymuses*: The Washington University in St. Louis School of Medicine Institutional Review Board reviewed and approved Marina Cella’s study for the collection of thymuses from children under 17 years of age undergoing cardiac surgery (IRB approval number 202009010). *(2) Adult thymuses*: The Washington University in St. Louis School of Medicine Institutional Review Board reviewed and approved a study for Understanding healthy immune aging in peripheral tissues (IRB approval number 202402173).

## METHOD DETAILS

### Cell isolation

#### PBMCs

##### Healthy samples:

PBMCs were isolated from whole blood by density gradient centrifugation using Histopaque-1077 (Sigma, 10771) according to the manufacturer’s instructions. Briefly, the whole blood was diluted in a 1:1 ratio with DPBS (Gipco,14190136) with 2mM EDTA (Corning, 46–034-CI). The diluted blood was overlaid on Histopaque-1077 and centrifuged at 500g for 30 min at room temperature without breaks. PBMC were collected from diluted plasma - Histopaque interface and washed twice in DPBS–2mM EDTA. PBMC were cryopreserved in CryoStor CS10 freezing medium (Biolife Solutions, 210502) and stored at –80°C. *Influenza samples*: Peripheral blood samples were obtained into ethylenediaminetetraacetic acid-anticoagulated tubes (BD Biosciences), prepared within 8 hours of phlebotomy into peripheral blood mononuclear cells using Ficoll density gradient purification, live cells were counted, and aliquots were frozen and stored in the vapor phase of liquid nitrogen prior to analysis in media containing 40% fetal bovine serum/60% RPMI 1640 medium/10% dimethyl sulfoxide. *Atherosclerosis samples*: Peripheral blood from coronary artery disease subjects as well as subjects who had undergone cardiac catheterization to exclude CAD was drawn into BD K2 EDTA vacutainer tubes and processed at room temperature (RT) within one hour of collection. PBMCs were isolated by Ficoll-Paque PLUS (GE Healthcare Biosciences AB) gradient centrifugation using SepMate-50 tubes (Stemcell) following the manufacturer’s protocol. Trypan blue staining of PBMCs was performed to quantify live cell counts. PBMCs were cryopreserved in freezing solution (90% FBS with 10% DMSO). PBMC vials were stored in Mr. Frosty (Thermo Fisher) for 48 hours at −80 °C and were then stored in liquid nitrogen until use.

#### Thymocytes

Thymocytes were isolated from approximately 1cm^2^ of the lower part of the thymus lobe. Samples were cut into pieces with scissors and morselized against a 100mm filter, followed by washing with DPBS–2mM EDTA. Thymocytes were cryopreserved in CryoStor CS10 freezing medium (Biolife Solutions, 210502) and stored at –80°C.

### Multiome-seq of naive T cells

Naive CD8^+^ and CD4^+^ T cells from two donors (Table S2) were sorted using Aurora Cell Sorter (Cytek Biosciences), and cells were pooled equally into one sample. Permeabilized nuclei for multiome-seq were prepared according to Nuclei Isolation for Single Cell Multiome ATAC + Gene Expression Sequencing protocol for PBMCs (10x Genomics). scRNA/ATAC-seq libraries were constructed and sequenced at the McDonnell Genome Institute (WASHU, St Louis) using Illumina NovaSeq X plus 10B flow cells, targeting 70,000 read for pair/nucleus GEX, 40,000 reads for pair/nucleus ATAC.

### RNA-seq of CD38 subsets

Naive CD8^+^ and CD4^+^ T cells were sorted into 3 subsets based on their expression intensity of CD38 using Aurora Cell Sorter (Cytek Biosciences). A negative fraction was set up using FMO CD38 control; CD38^hi^ fraction was gated using SP4 and SP8 thymocytes as a positive control. mRNA was isolated directly from cell pellets with RNeasy Plus Micro Kit (QIAGEN, 74034). RNA-seq libraries were constructed and sequenced at the McDonnell Genome Institute (WASHU, St Louis) using the Takara-Clontech SMARTer system and Illumina NovaSeq6000 S4 XP flow cells with 2×150 paired-end reads.

### Naive cell sorting and stimulation conditions

Lymphocytes from young and old donors (CD16^−^CD14^−^ PBMCs enriched from PBMC by negative selection using magnetic beads; Miltenyi Biotec; Table S2) were sorted into naive CD8^+^ and CD4^+^ T cell subsets using Aurora Cell Sorter (Cytek Biosciences). Correspondingly, PBMC isolated from young donors (Table S2) were sorted into CD38 subsets of naive CD8^+^ and CD4^+^ T cells. Namely, CD38^−^ and CD38^+^ naive CD8^+^ T cells and CD38^lo^ and CD38^hi^ naive CD4^+^ T cells. The used antibodies are specified in Table S5. Sorted cells were cultured in RPMI 1640 medium supplemented with 10% FBS, 1% penicillin/streptomycin, and 2mM L-glutamine in concentration 0.3×10^5^ cells/ml in a V-shape 96-well plate. (1) Naive CD8^+^ and CD4^+^ T cells were stimulated for 6 hours with 10 ng/ml PMA, 500 ng/ml ionomycin, and 1x Brefeldin A at 37°C, 5% CO_2_. Unstimulated cells were incubated with 1x Brefeldin A to capture baseline production of cytokines. After incubation, cells were processed by standard flow cytometry staining for expression of cytokines. The used antibodies are specified in Table S5. Flow cytometry data were acquired using Cytek Aurora cytometer (Cytek Biosciences) equipped with 5 lasers, followed by spectral unmixing using SpectroFlo software (Cytek Biosciences). Unmixed results were analyzed using FlowJo V 10.10.0 software (Tree Star). (2) Naive CD8^+^ and CD4^+^ T cells were stimulated for 24 hours with anti-CD3 and anti-CD28 DynaBeads (bead-to-cell ratio 1:2; Gibco, 11161D). Cell supernatants were collected and analyzed for cytokine levels by multiplex fluorescent bead assay (Eve Technologies, HDF15 assay). Cells were used for the isolation of mRNA using RNeasy Plus Micro Kit (QIAGEN, 74034). RNA-seq libraries were constructed and sequenced at the McDonnell Genome Institute (WASHU, St Louis) using the Takara-Clontech SMARTer system and Illumina NovaSeq6000 S4 XP flow cells with 2×150 paired-end reads.

### TREC content analysis

Naive T cells from young donors (Table S2) were enriched from PBMC by negative selection using magnetic beads (StemCell, #17961) and sorted into CD38^−/+/++^ subsets of naive CD8^+^ and CD4^+^ T cells using Aurora Cell Sorter (Cytek Biosciences). The isolated cells were lysed in 100 mg/L proteinase K (Sigma, P4850–1ML) for 1.5h at 56°C followed by 10 min at 95°C. The number of TRECs in 50,000 cells was determined by real-time quantitative PCR using the CFX Connect Real-Time PCR Detection System (Bio-Rad) and TaqMan system (Applied Biosystems), as described previously.^65,105^ Primers for signal-joint TREC were as follows: forward primer: 5’-CACATCCCTTTCAACCATGCT-3’, reverse: 5’-GCCAGCTGCAGGGTTTAGG-3’ (IDT); and TaqMan probe FAM-ACACCTCTGGTTTTTGTAAAGGTGCCCACT-TAMRA (ThermoFisher Scientific). The number of TREC was quantified using standard curves generated by using TREC plasmid (GeneArt^™^ Construct ID 18ACJFZP).

### Spectral flow cytometry

Cryopreserved PBMC were thawed and stained with Live/dead fixable blue or green dead cell stain kit (Invitrogen, L34962, L34969), followed by human TruStain FcX blocking solution (Biolegend, 422302). According to previous reports,^106,107^ chemokine receptors and γδTCR show decreased resolution between positive and negative signals in multicolor panels, so we use the sequential staining protocol to improve the resolution for these markers as we described previously.^7^ PBMC were pre-stained with a cocktail of resolution-sensitive markers for 5–10 min, followed by a direct addition of surface antibody cocktail, and incubated for an additional 30 min at 4 °C. For intracellular staining, Foxp3 /Transcription Factor Staining Buffer Set (eBioscience, 00–5523-00) was used according to the manufacturer’s instruction. The used antibodies are specified in Table S5. Flow cytometry data were acquired using Cytek Aurora cytometer (Cytek Biosciences) equipped with 5 lasers, followed by spectral unmixing using SpectroFlo software (Cytek Biosciences).

### Proliferation assay

Naive T cells from 36–43yo donors (n=7; Table S2) were enriched from PBMC by negative selection using magnetic beads (StemCell, #17961). Enriched naive T cells were stained using 2.5mM Trace Cell Violet dye according to the manufacturer’s instruction (Invitrogen, C34557) and sorted for CD38^+^ naive CD8^+^ T cells and CD38^hi^ naive CD4^+^ T cells using Aurora Cell Sorter (Cytek Biosciences). Sorted cells were cultured in RPMI 1640 medium supplemented with 10% FBS, 1% penicillin/streptomycin, and 2mM L-glutamine in concentration 0.5×10^5^ cells/ml in a V-shape 96-well plate with IL-7 (10ng/ml, Biolegend, 581902) and IL-15 (10ng/ml, Biolegend, 570302) for 10 days at 37°C, 5% CO_2_. On days 3 and 6, the medium was replaced by a fresh RPMI medium with cytokines. On day 10, cells were stained by antibodies (Table S5) for RTE markers, as described above, and analyzed using a Cytek Aurora cytometer (Cytek Biosciences) equipped with 5 lasers.

### Spectral flow cytometry analysis of aging cohort

#### Spectral cytometry data preprocessing

Unmixed spectral data were transformed from range −10^7^..10^7^ to range −10..10 with *transFlowVS* function from R package *flowVS*^98^ v2.2.0, using asinh(x/cofactor) with cofactor equal to 3000. Each file was subsampled to 100,000 events with *sample* function from R base package^72^ v4.0.3 to reduce the computing resource requirements and balance the data contribution across the samples. Donors with less than 100,000 events were not included into analysis.

#### Cell type identification

Quality control and cell type identification procedures were applied in 3 steps: (a) UMAP construction, (b) Leiden clusterization, and (c) cell selection. UMAP construction for the selected markers was created by the *UMAP* function of R package *uwot*^99^ v0.1.10 on scaled and centered expression matrix using *scale* function from R base package^72^ v4.0.3. For Leiden clusterization multiple sub-steps were done: (a) k nearest neighbors determining for each event using *hnsw_knn* function with squared Euclidean parameter from R package *RcppHNSW*^100^ v0.3.0, (b) calculating nearest-neighbor distances using functions *rcpp_parallel_jce* and *dedup_links* from R package *FastPG*^101^ v0.0.8, (c) Leiden clustering using JAVA library CWTSLeiden/networkanalysis^102^ v1.1.0 and its command-line tool RunNetworkClustering with Modularity and weighted-edges parameters. Parameters k and resolution for Leiden clustering were selected for each launch individually (exact values for each step can be found in a GitHub repository https://github.com/JetBrains-Research/cd38-in-cd8-cd4-naive-cytek/blob/main/expression_processing/expression_processing.qmd). The cell selection on each procedure run was determined according to the expression of the markers for each cluster. The clustering pipeline and cell selection procedure were applied hierarchically, to subsequently subset only naive CD8^+^ and CD4^+^ T cells. Specifically, cells were filtered in the order below:

1. Debris, doublets, and dead cell filtering - All events and all technical markers (FSC, SSC, LIVE DEAD and AF) were used for clustering to determine debris, doublets, and dead cells according to FSC and SSC features and high LIVE DEAD expression; The clustering procedure was repeated twice to access the quality of filtering.
2. T cells selection – Clustering of cells defined in (1) using 8 markers (CD3, CD4, CD8, CD14, CD19, CD56, CD25, CD45RA) - selecting clusters with high CD3 marker expression;
3. CD8^+^ T cells and CD4^+^ T cells selection - Clustering of CD3 cell defined in (2) using 8 markers (CD3, CD4, CD8, CD14, CD19, CD56, CD25, CD45RA) - selecting two groups of clusters - with high CD8 marker expression and high CD4 marker expression;
  (4_1) Naive CD8^+^ T cells selection – Clustering of CD8^+^ cells as defined in (3) using 18 markers (Fas, CD45RA, KLRG1, Cx3CR1, GzmB, CD8, GzmK, CD27, CD28, CD56, TRAV2, γδTCR, CCR7, NKp80, CD31, CD4, CCR4, CD159c) - selecting clusters with high CD45RA, CCR7 and low Fas markers expression (naive CD8^+^ T cells);
  (4_1a) Naive CD8+ T cells clustering - Clustering of naive CD8^+^ cells defined in (4_1) using 4 markers (CD38, CD25, CD27, CXCR3) - final CD8 naive expression matrix for statistics calculation and figures visualization;
  (4_2) CD4+ T cells filtering – Clustering of CD4^+^ cells as defined in (3) using 10 markers (CD4, Fas, CD45RA, CD8, Cx3CR1, γδTCR, CD56, CD14, CD19, CD3) - removing clusters of γδT cells (γδTCR^+^), cytotoxic cells (KLRG1^+^CD57^+^GzmB^+^CD27^−^);
  (4_2a) naive CD4+ T cells selection – To select CD45RA^+^Fas^−^ cells within the CD4^+^ T cell subset, we used the “gating threshold construction” approach. This method enabled us to accurately identify and isolate cells among various intermediate values of Fas and CD45RA markers, which are specific for CD4^+^ T cells. CD45RA and Fas threshold constructions were performed individually for each sample using Gaussian fitting of their densities with *nlsLM* function from R package *minpack.lm*^103^ v1.2–1. Appling 2 steps: (a) Fitting CD45RA density of CD4^+^ filtered cells with two Gaussians and calculating their intersect using *uniroot* function from R package *stats*^72^ v4.0.3, (b) Making threshold for Fas by fitting Fas density of CD4^+^ filtered cells with CD45RA higher then intersect value from (a) with one gaussian and taking as a threshold μ + σ (Gaussian expected value + Gaussian deviation). Cells with CD45RA value above the threshold and Fas below the threshold are considered as naïve CD4^+^ T cells;
  (4_2b) Treg cell removal – Clustering of CD45RA^+^Fas^−^ CD4^+^ cells as defined in (4_2a) using 8 markers (CD4, CD3, CD45RA, CXCR3, Fas, CD25, CD127, CD31) removing cluster of Tregs (CD25 high expression and CD127 low expression);
  (4_2c) conventional naive CD4^+^ T cells – Clustering of conventional naive CD4^+^ cells as defined in (4_2b) using 4 markers (CD38, CD25, CD27, CXCR3) - final CD4^+^ naive expression matrix for statistics calculation and figures visualization.

During the naive T cell selection steps, clusters that did not belong to T cells (~0.1%) and any residual cells from earlier steps were considered technical contaminants and were filtered out. Sample E06 was considered a technical outlier and was removed from the further analysis after the clustering procedure due to insufficient number of naive CD8^+^ T cells to achieve the appropriate statistical power.

The selection of subpopulations inside naive T cells was based on the subsequent manual threshold selection. First, all naïve T cells with CXCR3 values higher than the threshold were defined as CXCR3^+^ mature cells. After CXCR3^+^ cell sub-setting, the procedure was repeated for CD38 threshold defining CD38^++^ cells and subsequently for CD25 defining CD25^lo^ mature cells. Leftover cells were considered as mature naïve T cells. Thresholds values can be found in a GitHub repository https://github.com/JetBrains-Research/cd38-in-cd8-cd4-naive-cytek/blob/main/visualization/visualization.qmd.

Data for Fas MFI median boxplot was calculated both for CD8^+^ and CD4^+^ cells with *median* function from R package *stats*^72^ v4.0.3. As input, we used Fas values coming from CD8^+^/CD4^+^ naive and memory T cell populations from each sample. Memory T cells defined for CD8^+^ cells are all clusters not selected on step (4_1) without cells with high expression of γδTCR and TRAV2. Memory T cells defined for CD4^+^ cells are all clusters not selected on step (4_2a) without cells with high expression of γδTCR and CD25.

### Visualization of aging cohort cytometry data

UMAP plots for markers expression are created using functions *geom_point* from R package *ggplot2*^95^ v3.3.3 and *geom_point_rast* from R package *ggrastr*^104^ v0.2.1 with negative values of expression considered as zero and values higher than 99th percentile equal to it. Plot visualizing the age of donors was constructed using function *geom_density* from R package *ggplot2*^95^ v3.3.3. Boxplots showing cell subset proportions among age groups were constructed using the function *geom_boxplot* from R package *ggplot2*^95^ v3.3.3. Pairwise statistic plots were created using the function *geom_raster* from the R package *ggplot2*^95^ v3.3.3.

### Single-cell RNA-sequencing analysis for PBMC datasets

#### Accession of the datasets

Preprocessed single-cell objects with CD8^+^ and CD4^+^ T cells from Terekhova, Swain, Bohacova et al.^7^ were previously generated in our laboratory and publicly accessible through Synapse, accession no: syn49637038. Preprocessed single-cell objects with human CD8^+^ and CD4^+^ T cells from Mogilenko et al.^20^ were previously generated in our laboratory and publicly accessible through Synapse, accession no: syn22255433.

#### Naive T cell selection

We utilized the clustering definitions provided in the original papers to select the clusters from CD4^+^ and CD8^+^ T cell datasets. Specifically, we selected clusters characterized by the expression of *CCR7* and *SELL* genes, along with high levels of CD45RA and CD62L surface proteins, while lacking CD45RO expression (original cluster names from Mogilenko et al.^20^: CD4 Tn cells, CD8 Tn cells; from Terekhova et al.^7^: CD4 Naive, CD8 Naive). Clusters, which exhibited IFN-signaling signature (Naive-IFN) were not included in the study.

Selected data underwent the new clustering pipeline as described below. After the first round, clusters expressing memory phenotype markers (CCL5 gene, CD45RO surface protein), along with cytotoxic markers (granzyme genes, GNLY, NKG7), were excluded from the analysis. Subsequently, the clustering pipeline was rerun, beginning with the redefinition of variable features.

#### Clustering pipeline

Log-normalized data from objects was used for downstream analysis. Analysis was performed inside *R* environment^72^ 4.0.2 using *Seurat* package^73^ v4.0.5. Cells with less than 20 percent of reads belonging to ribosomal genes were filtered out. Variable features were selected based on average expression and dispersion with cut-offs to leave ~1,500 variable features (upper cutoff: Inf; lower cutoffs: Terekhova et al.^7^ CD4^+^ cells: 0.01 for mean and 0.57 for dispersion; CD8^+^ cells: 0.01 for mean and 0.7 for dispersion; Mogilenko et al.^20^ CD4^+^ cells: 0.1 for mean and 0.85 for dispersion; CD8^+^ cells: 0.01 for mean and 1.1 for dispersion). Genes encoding alpha, beta TCR chains, and MHC class alleles were excluded from the variable features. Expression values coming from variable genes were scaled and centered. Variations coming from the total number of counts, percentage of counts belonging to mitochondrial genes, and sequencing batch were regressed out inside ScaleData function. UMAP dimensional reduction and shared nearest neighbor graph were calculated on 15 principal components with Euclidean metric parameter. For the objects coming from Terekhova, Swain, Bohacova et al.^7^ the number of neighbors for the graph was set to 15; from Mogilenko et al.^20^ - as default. Final clustering was defined based on resolution 0.2. For visualization, two SOX4^−^ clusters inside CD4^+^ T cells from Mogilenko et al.^20^ were merged together.

#### Pseudobulk generation and differential expression

Pseudobulk data were created by summing the raw count expression matrix for each donor inside the condition of interest. For single-cell data from Terekhova, Swain, Bohacova et al.^7^ only samples from the first visit of each donor were used. Replicates (donor + condition of interest) in naive T cell datasets with less than 10 cells were removed from the pseudobulk matrix. The *DESeq2* computational pipeline^74^ v1.30.1 was used to normalize counts and perform differential expression analysis. Genes with an average normalized expression of less than 2 were filtered. To fit dispersions to the mean intensity ‘local’ type was used. Differential expression between SOX4^−^ and SOX4^+^ clusters was accessed inside the A age group (<35 years old). For old vs young comparisons, A (<35 years old) and E (65+ years old) age groups were used. Differential expression for surface protein markers was performed on the centered log ratio transformation normalized assay using FindMarkers function from *Seurat* package^73^ v4.0.5.

### Single-cell RNA-sequencing analysis of the thymus dataset

Preprocessed single-cell object with human thymus cells from Park et al.^25^ was downloaded through a web portal (https://developmentcellatlas.ncl.ac.uk). Data read was performed using the readH5AD function from zellkonverter package^75^ v1.13.2 and the normalized matrix together with metadata was loaded inside the Seurat object. Subsetting specific single-positive populations was based on the original labels provided by Park et al.^25^ Differentially expressed genes between adult naive vs memory subsets were assessed using FindMarkers function from *Seurat* package73 v4.0.5 with logfc.threshold 0.01 and min.pct 0.01 parameters. Genes were subsetted to 9,000 the most expressed one, and gene set enrichment analysis was performed as described below (Enrichment analysis section). For enrichment score calculation inside each cell addGesecaScores function from fgsea package^76^ v1.25.2 was used.

### Single-nuclei multiome analysis

*Cell Ranger arc* pipeline v2.0.2 (available at 10x website) was applied for read alignment to reference genome GRCh38 (GENCODE v32/Ensembl 98), quantification of gene expression and peak accessibility in single nucleus. To demultiplex individual donor samples, *souporcell* pipeline v2.0^77^ was used. Only non-doublet and assigned cells were left for further analysis. Downstream single-cell analysis was performed using *Signac* package^78^ v1.7.0. Cells with a number of read counts of more than 75,000 or less than 8,000 for chromatin and of more than 10,000 for RNA data were filtered out. Additionally, we removed cells: having less than 450 genes; transcriptional start site enrichment score of less than 1; and a percentage of mitochondrial counts of more than 40. Single-nuclei GEX portion of the data was log-normalized and 2,000 variable features based on vst method were used to perform PCA. For transcriptional portion of data, genes encoding alpha, beta TCR chains, and MHC class alleles were excluded from the variable features. For ATAC-seq portion of the data peak calling was performed using *MACS2* tool^79^ implemented within *Signac* package^79^with default parameters. Next, chromatin data was normalized using term frequency inverse document frequency (TF-IDF) method and for SVD algorithm variable features with > 5 total counts were used. Both RNA and ATAC data were integrated independently using harmony package^80^ v1.2.0. UMAP coordinates were calculated using knn-graph built on both harmonized modalities using function FindMultiModalNeighbors from *Signac* package.^78^ PCA and SVD components were 1:25 and 2:25, respectively. Graph-based clustering was performed on the multimodal data using resolution 0.1, which resulted in 3 clusters: CD4-expressing cells, CD8-expressing cells, and doublets, expressing both of the markers. Doublets were filtered out from the data and next steps were proceed on the individual datasets for CD4 and CD8-expressing cells. Variable features were redefined inside each of the populations. For gene expression data 1,500 variable features were chosen with vst method and for chromatin portion of data top features with > 5 total counts were used. For transcriptional portion of data, genes encoding alpha, beta TCR chains, and MHC class alleles were excluded from the variable features. Integration and clustering algorithms were recalculated inside each of the populations with pipeline described above. For CD4^+^ T cells PCA and SVD components for FindMultiModalNeighbors function were 1:15 and 2:10, respectively. For CD8^+^ T cells PCA and SVD components for FindMultiModalNeighbors function were 1:11 and 2:13, respectively. Graph-based clustering was performed on the multimodal data using resolution 0.2. Peaks were redefined using the cluster grouping information with the peak calling method described above. Differential peaks were defined using logistic regression framework using FindAllMarkers function with 0.01 for logfc.threshold and min.pct parameters, and ‘nCount_peaks’ parameter as variables to test. For the motif enrichment, *JASPAR* 2024 database^108^ was downloaded from the official website (https://jaspar.elixir.no/). Homo sapience species, CORE collection with the latest version of motifs were used. To create a HOMER-compatible motif file dumpJaspar function from *monaLisa* package^109^ v1.9.0 was used. The *JASPAR* database^108^ was used for both *de novo* and known motif enrichment performed using *HOMER* tool^81^ v4.11 with findMotifsGenome.pl function with 200 bp region size parameter and masked hg38 genome and 1000 differentially accessible peaks as an input.

For track visualization, cellranger bam files were separated into multiple files corresponding to individual clusters using *samtools* package^82^ v1.2–242-g4d56437. Bam files were further sorted and indexed using *samtools* package.^82^ BigWig files were created using bamCoverage command from deeptools package^83^ v3.5.2 with CPM normalization parameter. Intersect of peaks was calculated using *bedtools*^84^ intersect function v2.30.0. The *WashU epigenome browser*^85^(https://epigenomegateway.wustl.edu/browser/) was used for the visualization of tracks.

### Bulk RNA-seq analysis

Pair-end reads coming from bulk RNA-seq of CD38 subsets and naive cell stimulation experiments were aligned with *STAR*^86^ v2.7.0f. Aligned reads were checked for strand specificity with infer_experiment.py script v2.6.4 from *RSeQC* package.^87^ RNA alignment metrics were assessed with CollectRnaSeqMetrics function from *Picard*^88^ v2.21.1. Gene counts were quantified using *featureCounts*^89^ v2.0.0 with no strand specificity parameter. Alignment and gene counts were generated against the GRCh38.p14 (GENCODE release 44) genome assembly. The batch effect coming from individual patients was removed with ComBat-seq function from sva package^90^ v3.38.0. The *DESeq2* computational pipeline^74^ v1.30.1 was used to normalize batch-corrected counts and perform differential expression analysis. Genes with less than 10 counts were filtered out. The design formula included conditions of interest (cell subsets) and replicate. DESeq analysis was performed with default parameters.

### Analysis of publicly available bulk RNA-seq and microarray data

Bulk RNA-seq data from van den Broek, Delemarre, Janssen et al.^17^ was accessed through the GEO database, accession no: GSE72400. The *DESeq2* computational pipeline^74^ v1.30.1 was used to normalize raw counts and perform differential expression analysis. Only naive T cell samples were used as an input. Genes with less than 10 counts were filtered out. The design formula included conditions of interest (cell subset and health status). DESeq analysis was performed with default parameters.

Normalized microarray datasets from Lee et al.^30^ (GEO database accession no: GSE1460) and from de Simone, Mazza et al.^43^ (GEO database accession no: GSE125102) were accessed using phantasus web-application.^93^ Normalized microarray data from Pekalski, Marcin et al.^57^ was downloaded from ArrayExpress database (accession no: E-MTAB-4853), probe names were converted to gene symbol nomenclature with *biomaRt* package^91^ v2.46.3, final matrix was normalized using rma function from *oligo* package^92^ v1.58.0 and uploaded inside *phantasus* web-application.^93^ Differential expression for microarray datasets between conditions of interest was performed within the phantasus web-application,^93^ using *limma* method.^110^

### Enrichment analysis

Gene set enrichment analysis was performed on the pre-ranked list of genes using the R package fgsea^76^ v1.25.2. For pseudobulk and bulk RNA-seq data ranking was performed based on Wald statistic from *DESeq2*^74^ package; for microarray data – based on t-statistic from *limma*^110^ package; for single-cell data – based on log_2_ fold-change from FindMarkers function. Signatures (gene sets) for enrichment analysis consisted of the top 100 genes from the pre-ranked list from comparisons of interest. Pathway enrichment was performed using HALLMARK and GO pathway database collection^111–113^ accessed through *msigdb* R package^94^ v7.2.1. Single-cell scatter plots showing signature profiles were plotted using plotCoregulationProfileReduction function from fgsea package v1.25.2. Enrichment plots were created with plotEnrichment function from fgsea package v1.25.2.

### Visualization of transcriptional data

Bar plots, volcano plots, scatter plots and box plots were created using *ggplot2* package^95^ v3.3.2. Volcano plots for bulk and pseudobulk data were visualized after shrinkage of effect size with a normal estimator inside *DESeq2* package pipeline^74^ v1.30.1. Heatmaps were created using *pheatmap* package^96^ v1.0.12 with row scaling. Density plots were created using contourPlot function from the *scToolkit* package (https://github.com/kevinblighe/scDataviz).

## QUANTIFICATION AND STATISTICAL ANALYSIS

All applied tests for the next-generation sequencing data and spectral cytometry data of 158 aging cohort were nonparametric, which does not require assumption on data distribution, and was conducted within the R environment^72^ 4.0.2. The name and type of the statistical tests are stated in the Figure legends. Post-hoc Dunn’s test was calculated using *dunnTest* function from FSA package^97^ v0.9.4.

Other comparisons were calculated in GraphPad Prism 10.1.0 software, the name and type of the statistical test are stated in the Figure legends. Data distribution was assumed to be normal.

In box plots representation horizontal bars showing the median value, hinges mark the 25th and 75th percentiles, whiskers cover values within 1.5 times the IQR from the hinges.

## Supplementary Material

mmc5

mmc1

mmc4

mmc6

mmc7

mmc8

mmc2

mmc3

SUPPLEMENTAL INFORMATION

Supplemental information can be found online at https://doi.org/10.1016/j.immuni.2024.08.019.

## Figures and Tables

**KEY RESOURCES TABLE:** 

REAGENT or RESOURCE	SOURCE	IDENTIFIER

Antibodies		

Brilliant Violet 711^™^ anti-human CD196 (CCR6), clone G034E3	BioLegend	Cat# 353435; RRID:AB_2629607
PE/Dazzle^™^ 594 anti-human CD197 (CCR7), clone G043H7	BioLegend	Cat# 353236, RRID:AB_2563641
Brilliant Violet 605^™^ anti-human CD103 (Integrin αE), clone Ber-ACT8	BioLegend	Cat# 350218, RRID:AB_2564283
APC anti-human CD127 (IL-7Rα), clone A019D5	BioLegend	Cat# 351316, RRID:AB_10900804
Spark Blue^™^ 550 anti-human CD14, clone 63D3	BioLegend	Cat# 367147, RRID:AB_2820021
Brilliant Violet^™^ 711 anti-human CD200 (OX2), clone OX-104	BioLegend	Cat# 329222, RRID:AB_2715823
Spark NIR^™^ 685 anti-human CD25, clone M-A251	BioLegend	Cat# 356151, RRID:AB_2888804
PE anti-human CD25, clone M-A251	BioLegend	Cat# 356103, RRID:AB_2561860
Brilliant Violet 750^™^ anti-human CD28, clone CD28.2	BioLegend	Cat# 302969, RRID:AB_2876593
Brilliant Violet 510^™^ anti-human CD3, clone SK7	BioLegend	Cat# 344827, RRID:AB_2563703
Pacific Blue^™^ anti-human CD3, clone SK7	BioLegend	Cat# 344824, RRID:AB_2563422
Brilliant Violet 605^™^ anti-human CD31, clone WM59	BioLegend	Cat# 303121, RRID:AB_2562148
APC/Fire^™^ 810 anti-human CD38, clone HB-7	BioLegend	Cat# 356643, RRID:AB_2860936
PE/Fire^™^ 810 anti-human CD38, clone S17015F	BioLegend	Cat# 397225, RRID:AB_2894562
PE/Fire^™^ 810 anti-human CD39, clone A1	BioLegend	Cat# 328245, RRID:AB_2894563
PerCP/Cyanine5.5 anti-human CD4, clone A161A1	BioLegend	Cat# 357414, RRID:AB_2565666
PerCP/Fire^™^ 806 anti-human CD4, clone SK3	BioLegend	Cat# 344693, RRID:AB_2941523
PE/Fire^™^ 640 anti-human CD45RA, clone HI100	BioLegend	Cat# 304169, RRID:AB_2876598
Brilliant Violet 570^™^ anti-human CD56 (NCAM), clone 5.1H11	BioLegend	Cat# 362539, RRID:AB_2565917
PE/Cyanine7 anti-human CD56 (NCAM), clone 5.1H11	BioLegend	Cat# 362509, RRID:AB_2563926
PerCP/Cyanine5.5 anti-human CD57, clone HNK-1	BioLegend	Cat# 359621, RRID:AB_2565929
Brilliant Violet 421^™^ anti-human CD62L, clone DREG-56	BioLegend	Cat# 304827, RRID:AB_10896429
PerCP anti-human CD8, clone SK1	BioLegend	Cat# 344707, RRID:AB_1967122
PE/Cyanine7 anti-human CD9, clone HI9a	BioLegend	Cat# 312115, RRID:AB_2728255
FITC anti-human CX3CR1, clone 2A9-1	BioLegend	Cat# 341606, RRID:AB_1626272
PE/Cyanine5 anti-human CD95 (Fas), clone DX2	BioLegend	Cat# 305610, RRID:AB_314548
APC/Fire^™^ 750 anti-human CD95 (Fas), clone DX2	BioLegend	Cat# 305637, RRID:AB_2629735
PE/Fire^™^ 700 anti-human TCR γ/δ, clone B1	BioLegend	Cat# 331237, RRID:AB_2876638
PE/Cyanine7 anti-human TCR γ/δ, clone B1	BioLegend	Cat# 331221, RRID:AB_2562890
PE/Dazzle^™^ 594 anti-human/mouse Granzyme B Recombinant, clone QA16A02	BioLegend	Cat# 372215, RRID:AB_2728382
Brilliant Violet 785^™^ anti-human HLA-DR, clone L243	BioLegend	Cat# 307641, RRID:AB_2561360
Brilliant Violet 605^™^ anti-human IFN-γ, clone B27	BioLegend	Cat# 506541, RRID:AB_2801101
PE/Fire^™^ 640 anti-human CD279 (PD-1), clone EH12.2H7	BioLegend	Cat# 329967, RRID:AB_2894474
PE/Cyanine7 anti-human TCR Vα7.2, clone 3C10	BioLegend	Cat# 351711, RRID:AB_2561993
Brilliant Violet 785^™^ anti-human TNF-α, clone MAb11	BioLegend	Cat# 502947, RRID:AB_2565857
PE anti-human LT-α (TNF-β), clone 359-81-11	BioLegend	Cat# 503105, RRID:AB_315275
BD OptiBuild^™^ BUV805 Mouse Anti-Human CD194, clone 1G1	BD	Cat# 752524, RRID:AB_2917514
BD OptiBuild^™^ BUV563 Mouse Anti-Human CD195, clone 2D7/CCR5	BD	Cat# 741401, RRID:AB_2870893
BD OptiBuild^™^ BUV737 Rat Anti-Human CCR7 (CD197), clone 3D12	BD	Cat# 741786, RRID:AB_2871135
BD OptiBuild^™^ BUV805 Mouse Anti-Human CCR9, clone C9Mab-1	BD	Cat# 752799, RRID:AB_2917779
BD Pharmingen^™^ APC Mouse Anti-Human CCR9 (CD199), clone C9Mab-1	BD	Cat# 567976, RRID:AB_2916803
BD Horizon^™^ APC-R700 Mouse Anti-Human CD127, clone HIL-7R-M21	BD	Cat# 565185, RRID:AB_2739099
BD Horizon^™^ BV650 Mouse Anti-Human CD127, clone HIL-7R-M21	BD	Cat# 563225, RRID:AB_2738081
BD OptiBuild^™^ BV786 Mouse Anti-Human CD159c (NKG2C), clone 134591	BD	Cat# 748170, RRID:AB_2872631
BD Horizon^™^ BUV496 Mouse Anti-Human CD16, clone 3G8	BD	Cat# 612944, RRID:AB_2870224
BD Horizon^™^ BV480 Mouse Anti-Human CD19, clone SJ25C1	BD	Cat# 566103, RRID:AB_2739505
BD Pharmingen^™^ APC-H7 Mouse Anti-Human CD27, clone M-T271	BD	Cat# 560223, RRID:AB_1645473
BD Horizon^™^ BUV805 Mouse Anti-Human CD3, clone SK7	BD	Cat# 612894, RRID:AB_2870182
BD Horizon^™^ BV421 Mouse Anti-Human CD4, clone RPA-T4	BD	Cat# 562424, RRID:AB_11154417
BD OptiBuild^™^ BUV395 Mouse Anti-Human CD45RA, clone 5H9	BD	Cat# 740315, RRID:AB_2740052
BD Horizon^™^ RB780 Mouse Anti-Human CD69, clone FN50	BD	Cat# 568755
BD OptiBuild^™^ BV510 Mouse Anti-Human CD93, clone R139	BD	Cat# 743196, RRID:AB_2741335
BD OptiBuild^™^ BUV615 Mouse Anti-Human CD183 (CXCR3), clone 1C6/CXCR3	BD	Cat# 751126, RRID:AB_2875154
BD OptiBuild^™^ BV650 Rat Anti-Human CXCR5 (CD185), clone RF8B2	BD	Cat# 740528, RRID:AB_2740238
BD Horizon^™^ PE-CF594 Mouse Anti-Human FoxP3, clone 236A/E7	BD	Cat# 563955, RRID:AB_2738507
BD Pharmingen^™^ Alexa Fluor^®^ 488 Armenian Hamster Anti-Helios, clone 22F6	BD	Cat# 563950, RRID:AB_2738505
BD Horizon^™^ BUV737 Rat Anti-Human IL-2, clone MQ1-17H12	BD	Cat# 612836, RRID:AB_2870158
BD Horizon^™^ BV510 Mouse Anti-Human IL-8, clone G265-8	BD	Cat# 563311, RRID:AB_2738132
BD Horizon^™^ R718 Mouse Anti-TCF-7/TCF-1, clone S33-966	BD	Cat# 567587, RRID:AB_2916656
BD OptiBuild^™^ BUV661 Mouse Anti-Human TCR Vα7.2, clone OF-5A12	BD	Cat# 750392, RRID:AB_2874562
Alexa Fluor^®^ 647 Rabbit Anti-Lef1, clone C12a5	Cell Signaling	Cat# 14022S
PE anti-human PTK7, clone 188B	Miltenui Biotec	Cat# 130-122-923
Human NKp80/KLRF1 Alexa Fluor^®^ 647-conjugated Antibody, clone 239127	R&D Systems	Cat# FAB1900R100
Super Bright^™^ 436 anti-human CD123, clone 6H6	ThermoFisher	Cat# 62-1239-42
PerCP-eFluor^™^ 710 anti-human GzmK, clone G3H69	ThermoFisher	Cat# 46-8897-42
PE-Cyanine7 anti-human KLRG1, clone 13F12F2	ThermoFisher	Cat# 25-9488-42
PE anti-TOX, clone TXRX10	ThermoFisher	Cat# 12-6502-82

Biological samples		

Human PBMCs, see Table S2	This paper	N/A
Human thymocytes	This paper	N/A

Chemicals, peptides, and recombinant proteins		

Human TruStain FcX^™^ 200 tests	BioLegend	Cat# 422302
Brilliant Stain Buffer Plus	BD	Cat# 566385
Brefeldin A	ThermoFisher	Cat# 00-4506-51
Ionomycin calcium salt from Streptomyces conglobatus	Sigma	Cat# I0634-1MG
Phorbol 12-myristate 13-acetate	Sigma	Cat# P8139-1MG
Dynabeads^™^ Human T-Activator CD3/CD28 for T Cell Expansion and Activation	ThermoFisher	Cat# 11161D
Recombinant Human IL-7 (carrier-free)	BioLegend	Cat# 581902
Recombinant Human IL-15 (carrier-free)	BioLegend	Cat# 570302
Proteinase K	Sigma	Cat# P4850-1ML

Critical commercial assays		

CryoStor CS10 Freeze Media	Biolife Solutions	Cat#210102
EasySep^™^ Human Naïve Pan T Cell Isolation Kit	StemCell	Cat# 17961
EasySep^™^ Human Naïve CD8+ T Cell Isolation Kit	StemCell	Cat# 19258
EasySep^™^ Human Naïve CD4+ T Cell Isolation Kit	StemCell	Cat# 19555
RNeasy Plus Micro Kit	QIAGEN	Cat# 74034
LIVE/DEAD Fixable Blue viability dye	Invitrogen	Cat# L34962
LIVE/DEAD Fixable Green viability dye	Invitrogen	Cat# L34969
CellTrace^™^ Violet Cell Proliferation Kit, for flow cytometry	Invitrogen	Cat# C34557
eBioscience^™^ Foxp3 / Transcription Factor Staining Buffer Set	ThermoFisher	Cat# 00-5523-00
Platinum^™^ Quantitative PCR SuperMix-UDG	ThermoFisher	Cat# 11730017

Deposited data		

Raw and processed single-nuclei multiome of CD4 and CD8 naive cells	This paper	Synapse: syn53238645
Raw and processed bulk RNA-seq of CD38 subsets	This paper	Synapse: syn53238645
Raw and processed bulk RNA-seq of naive CD4 and CD8 cell stimulation	This paper	Synapse: syn53238645
Processed scRNA-seq with CD8 and CD4 T cells (ABF300 project)	Terekhova et al.^7^	syn49637038
Processed scRNA-seq with CD8 and CD4 T cells	Mogilenko et al.^20^	syn22255433
Processed single-cell object with human thymus cells	Park et al.^25^	https://developmentcellatlas.ncl.ac.uk/
Bulk RNA-seq data of naive T cells after thymectomy	van den Broek et al.^17^	GEO: GSE72400
Normalized microarray dataset of fetal CD4 thymocytes	Lee et al.^30^	GEO: GSE1460
Normalized microarray dataset of human CD8 naive T cell subsets (CXCR3 sorting)	De Simone et al.^43^	GEO: GSE125102
Normalized microarray dataset of human naive CD4 T cell subsets (CD25 sorting)	Pekalski et al.^57^	ArrayExpress: E-MTAB-4853
Raw and processed spectral cytometry data	This paper	Synapse: syn53238646

Oligonucleotides		

Primer: TREC Forward: CACATCCCTTTCAACCATGCT	IDT	N/A
Primer: TREC Reverse: GCCAGCTGCAGGGTTTAGG	IDT	N/A

Recombinant DNA		

TaqMan probe FAM-ACACCTCTGGTTTTTGTAAAGGTGCCCACT-TAMRA	ThermoFisher	Cat# 450025
TREC plasmid	GeneArt	Construct ID 18ACJFZP

Software and algorithms		

SpectroFlo	Cytek Biosciences	https://cytekbio.com/pages/spectro-flo
FlowJo v10.10.0	Tree Star	https://www.flowjo.com/solutions/flowjo/downloads
Prism v10.1.0	GraphPad	https://www.graphpad.com/scientific-software/prism/
R v4.0.2 and v4.0.3	R Core Team^72^	https://cran.r-project.org/mirrors.html
Seurat v4.0.5	Hao et al.^73^	https://satijalab.org/seurat/articles/install
DESeq2 v1.30.1	Love et al.^74^	https://bioconductor.org/packages/release/bioc/html/DESeq2.html
zellkonverter v1.13.2	Zappia and Lun^75^	https://www.bioconductor.org/packages/release/bioc/html/zellkonverter.html
fgsea v1.25.2	Korotkevich et al.^76^	https://github.com/ctlab/fgsea
Cell Ranger Single-Cell Software Suite	10x Genomics	https://support.10xgenomics.com/single-cell-gene-expression/software/overview/welcome
souporcell pipeline v2.0	Heaton et al.^77^	https://github.com/wheaton5/souporcell
Signac v1.7.0	Stuart et al.^78^	https://cran.r-project.org/web/packages/Signac/index.html
MACS2	Zhang et al.^79^	https://github.com/macs3-project/MACS
Harmony v1.2.0	Korsunsky et al.^80^	https://github.com/immunogenomics/harmony
monaLisa v1.9.0	Machlab et al.^78^	https://www.bioconductor.org/packages/release/bioc/html/monaLisa.html
HOMER v4.11	Heinz et al.^81^	http://homer.ucsd.edu/homer/
samtools v1.2-242-g4d56437	Li et al.^82^	https://www.htslib.org/
deeptools v3.5.2	Ramirez et al.^83^	https://deeptools.readthedocs.io/en/develop/content/tools/bamCoverage.html
bedtools v2.30.0	Quinlan and Hall^84^	https://github.com/arq5x/bedtools2
Web-application WashU Epigenome Browser	Li et al.^85^	https://epigenomegateway.wustl.edu/
STAR v2.7.0f	Dobin et al.^86^	https://github.com/alexdobin/STAR
RSeQC (infer_experiment.py v2.6.4)	Wang et al.^87^	https://github.com/MonashBioinformaticsPlatform/RSeQC
Picard v2.21.1	Github^88^	https://github.com/broadinstitute/picard
featureCounts v2.0.0	Liao et al.^89^	https://subread.sourceforge.net/
sva v3.38.0	Leek et al.^90^	https://bioconductor.org/packages/release/bioc/html/sva.html
biomaRt v2.46.3	Durinck et al.^91^	https://bioconductor.org/packages/release/bioc/html/biomaRt.html
oligo v1.58.0	Carvalho and Irizarry^92^	https://bioconductor.org/packages/release/bioc/html/oligo.html
Web-application Phantasus v1.19.3	Kleverov et al.^93^	https://artyomovlab.wustl.edu/phantasus/
msigdb R v7.2.1	Dolgalev^94^	https://cran.r-project.org/web/packages/msigdbr/
ggplot2 v3.3.2 and v3.3.3	Wickham^95^	https://cloud.r-project.org/web/packages/rgeos/index.html
pheatmap v1.0.12	Raivo^96^	https://cran.r-project.org/web/packages/pheatmap/index.html
scToolkit v1.13.10	Github	https://github.com/kevinblighe/scDataviz
FSA v0.9.4	Ogle et al.^97^	https://cran.r-project.org/web/packages/FSA/index.html
flowVS v2.2.0	Azad et al.^98^	https://bioconductor.org/packages/release/bioc/html/flowVS.html
uwot v0.1.10	Melville et al.^99^	https://github.com/jlmelville/uwot
RcppHNSW v0.3.0	Melville^100^	https://github.com/jlmelville/rcpphnsw
FastPG v0.0.8	Bodenheimer^101^	https://github.com/sararselitsky/FastPG
networkanalysis v1.1.0	Traag et al.^102^	https://github.com/CWTSLeiden/networkanalysis
minpack.lm v1.2-1	Elzhov et al.^103^	https://cran.r-project.org/web/packages/minpack.lm/index.html
ggrastr v0.2.1	Petukhov et al.^104^	https://github.com/VPetukhov/ggrastr
Code to process and visualize spectral cytometry data	This paper	https://github.com/JetBrains-Research/cd38-in-cd8-cd4-naive-cytek/

Other		

Links for the single-cell online browser and interactive heatmaps		Synapse: syn53238645

## Highlights

- Human RTEs are epigenetically and transcriptionally defined by SOX4, IKZF2, and TOX
- Both CD8^+^ and CD4^+^ RTEs are characterized by high surface expression of CD38
- CD38 expression is a reliable RTE marker also under chronic and acute inflammation
- Naive T cells show an age-related decline in CD38^++^ RTEs and an increase in CXCR3^hi^ cells
