# Supplementary material for: Multidimensional profiling of human T cells reveals high CD38 expression, marking recent thymic emigrants and age-related naive T cell remodeling
Source: Immunity. Author manuscript; Available in PMC 2026 May 4. (PMC13138122; doi:10.1016/j.immuni.2024.08.019)
Supplement: mmc1 [file NIHMS2160335-supplement-mmc1.pdf]

Immunity, Volume *57*

Supplemental information

Multidimensional profiling of human T cells reveals high CD38 expression, marking recent thymic emigrants and age-related naive T cell remodeling

Pavla Bohacova, Marina Terekhova, Petr Tsurinov, Riley Mullins, Kamila Husarcikova, Irina Shchukina, Alina Ulezko Antonova, Barbora Echalar, Jan Kossl, Adam Saidu, Thomas Francis, Chelsea Mannie, Laura Arthur, Stephen D.R. Harridge, Daniel Kreisel, Philip A. Mudd, Angela M. Taylor, Coleen A. McNamara, Marina Cella, Sidharth V. Puram, Theo van den Broek, Femke van Wijk, Pirooz Eghtesady, and Maxim N. Artyomov

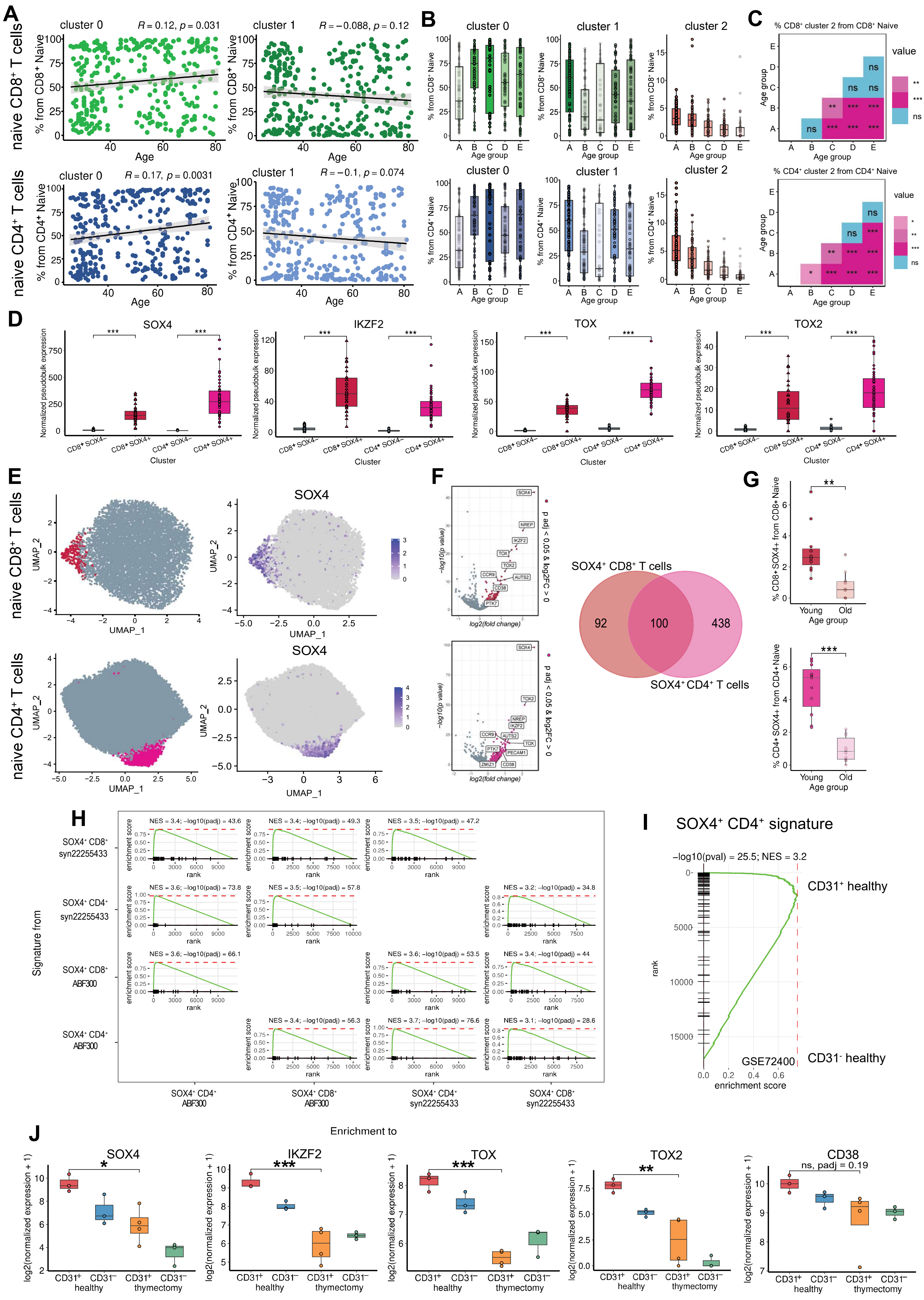

A
naive CD8+ T cells
naive CD4+ T cells
cluster 0 R = 0.12, p = 0.031
cluster 1 R = −0.088, p = 0.12
cluster 0 R = 0.17, p = 0.0031
cluster 1 R = −0.1, p = 0.074
% from CD8+ Naive
% from CD4+ Naive
Age
B
cluster 0
cluster 1
cluster 2
Age group
C
% CD8+ cluster 2 from CD8+ Naive
% CD4+ cluster 2 from CD4+ Naive
value
Age group
D
SOX4
IKZF2
TOX
TOX2
Normalized pseudobulk expression
CD8+ SOX4−
CD8+ SOX4+
CD4+ SOX4−
CD4+ SOX4+
Cluster
E
UMAP_1
UMAP_2
F
p adj < 0.05 & log2FC > 0
−log10(p value)
log2(fold change)
SOX4+ CD8+ T cells
SOX4+ CD4+ T cells
92
100
438
G
% CD8+SOX4+ from CD8+ Naive
% CD4+ SOX4+ from CD4+ Naive
Young
Old
Age group
H
Signature from
SOX4+ CD8+ syn22255433
SOX4+ CD4+ syn22255433
SOX4+ CD8+ ABF300
SOX4+ CD4+ ABF300
NES = 3.4; −log10(padj) = 43.6
NES = 3.4; −log10(padj) = 49.3
NES = 3.5; −log10(padj) = 47.2
NES = 3.6; −log10(padj) = 73.8
NES = 3.5; −log10(padj) = 57.8
NES = 3.2; −log10(padj) = 34.8
NES = 3.6; −log10(padj) = 66.1
NES = 3.6; −log10(padj) = 53.5
NES = 3.4; −log10(padj) = 44
NES = 3.4; −log10(padj) = 56.3
NES = 3.7; −log10(padj) = 76.6
NES = 3.1; −log10(padj) = 28.6
enrichment score
rank
SOX4+ CD4+ ABF300
SOX4+ CD8+ ABF300
SOX4+ CD4+ syn22255433
SOX4+ CD8+ syn22255433
Enrichment to
I
SOX4+ CD4+ signature
−log10(pval) = 25.5; NES = 3.2
CD31+ healthy
CD31- healthy
GSE72400
rank
enrichment score
J
SOX4
IKZF2
TOX
TOX2
CD38
ns, padj = 0.19
log2(normalized expression + 1)
CD31+ CD31− CD31+ CD31−
healthy thymectomy

Supplementary Figure 1: Characterization of naive T cell clusters and their validation, related to Figure 1.

(**A**) Scatterplot showing the percentage of clusters 0 and 1 from total naive $CD8^+$ (top) and $CD4^+$ (bottom) T cells against age. Black line represents the best-fitted linear regression, with the shading showing the 95% confidence intervals. R represents the Pearson correlation coefficient.

(**B**) Boxplots showing the percentage of corresponding clusters from total naive $CD8^+$ (top) and $CD4^+$ (bottom) T cells by the age group (n=317 overall (A=96, B=53, C=41, D=54, E=73).

(**C**) Heatmaps representing significance for pairwise comparisons of the $SOX4^+$ cluster percentages between age groups A–E, p-adj by post-hoc Dunn's test after one-way Kruskal–Wallis test with Holm correction method ($SOX4^+$ $CD4^+$ n=47; $SOX4^+$ $CD8^+$ n=30).

(**D**) Boxplots showing normalized pseudobulk expression of key transcription factors in $SOX4^+$ and $SOX4^-$ clusters of naive T cells, p-adj using Wald test from DESeq2 (n=317 overall (A=96, B=53, C=41, D=54, E=73)).

(**E**) UMAP plot of naive $CD8^+$ (top) and $CD4^+$ (bottom) T cells showing $SOX4^+$ and $SOX4^-$ clusters from publicly available data from syn22255433 (Mogilenko et al. [1]) (left). UMAP plots showing normalized expression for *SOX4* gene in naive $CD8^+$ and $CD4^+$ T cells from syn22255433 (Mogilenko et al. [1]) (right).

(**F**) Volcano plot for comparison of $SOX4^+$ vs $SOX4^-$ clusters in naive $CD8^+$ (top) and $CD4^+$ (bottom) T cells (left). Venn diagram showing differentially expressed gene overlap (p.adj<0.05) between $SOX4^+$ clusters in naive $CD4^+$ and $CD8^+$ T cells (right). syn22255433 dataset.

(**G**) Boxplots showing the percentage of $SOX4^+$ clusters of naive $CD8^+$ (top) and $CD4^+$ (bottom) T cells by the age group of the syn22255433 dataset two-sided Wilcoxon rank sum test (n=21).

(**H**) GSEA normalized enrichment score (NES) and p.adj values for pairwise signature enrichment from selected datasets to corresponding clusters.

(**I**) GSEA plots of single-cell $CD4^+$ $SOX4^+$ cluster signature enriched to the signature of $CD31^+$ naive $CD4^+$ T cells.

(**J**) Boxplots showing the normalized expression of selected genes in cell subsets of healthy and thymectomy individuals, source GSE72400 [2]. p-adj using Wald test from DESeq2

*p.adj<0.05, **p.adj<0.01, ***p.adj<0.001, ns=not significant

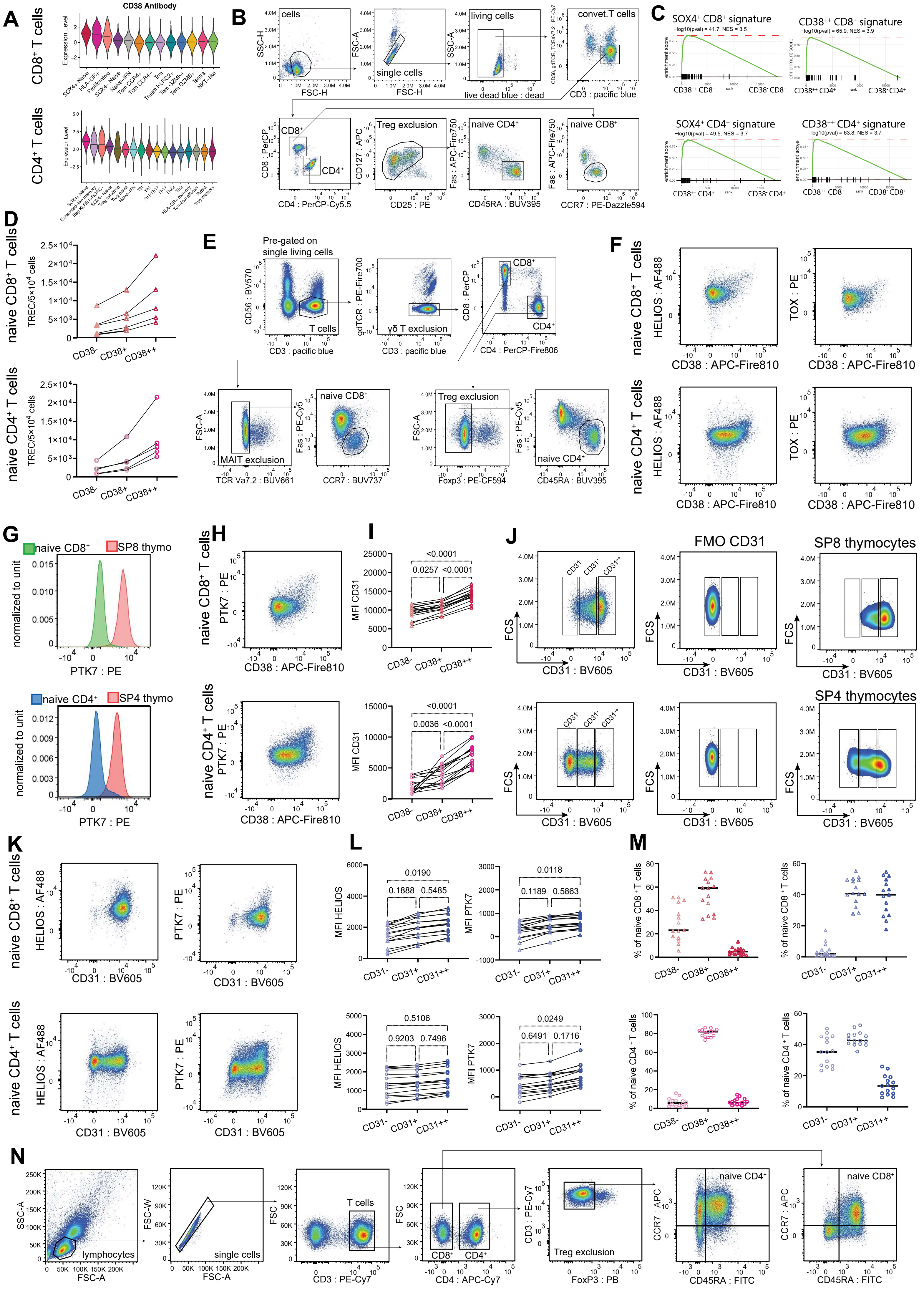

A
CD38 Antibody
CD8+ T cells
CD4+ T cells
Expression Level
SOX4+ Naive
HLA-DR+
Proliferative
SOX4- Naive
Naive-IFN
Tcm CCR4+
Tcm CCR4-
Trm
Temra
NKT-like
Treg naive
Th1
Th17
Th2
Terminal effector
Treg memory
B
cells
single cells
living cells
convet.T cells
SSC-H
FSC-H
FSC-A
SSC-A
live dead blue : dead
CD3 : pacific blue
CD56, gdTCR, TCRaV7.2 : PE-Cy7
CD8+
CD4+
CD8 : PerCP
CD4 : PerCP-Cy5.5
Treg exclusion
CD127 : APC
CD25 : PE
naive CD4+
Fas : APC-Fire750
CD45RA : BUV395
naive CD8+
CCR7 : PE-Dazzle594
C
SOX4+ CD8+ signature
−log10(pval) = 41.7, NES = 3.5
CD38++ CD8+ signature
−log10(pval) = 65.9, NES = 3.9
SOX4+ CD4+ signature
−log10(pval) = 49.5, NES = 3.7
CD38++ CD4+ signature
−log10(pval) = 63.8, NES = 3.7
enrichment score
rank
CD38++ CD8+
CD38- CD8+
CD38++ CD4+
CD38- CD4+
D
naive CD8+ T cells
naive CD4+ T cells
TREC/5×10^4 cells
CD38-
CD38+
CD38++
E
Pre-gated on single living cells
CD56 : BV570
T cells
CD3 : pacific blue
gdTCR : PE-Fire700
γδ T exclusion
CD8+
CD4+
CD8 : PerCP
CD4 : PerCP-Fire806
MAIT exclusion
FSC-A
TCR Va7.2 : BUV661
naive CD8+
Fas : PE-Cy5
CCR7 : BUV737
Treg exclusion
Foxp3 : PE-CF594
naive CD4+
CD45RA : BUV395
F
naive CD8+ T cells
naive CD4+ T cells
HELIOS : AF488
TOX : PE
CD38 : APC-Fire810
G
naive CD8+
SP8 thymo
naive CD4+
SP4 thymo
normalized to unit
PTK7 : PE
H
naive CD8+ T cells
naive CD4+ T cells
PTK7 : PE
CD38 : APC-Fire810
I
MFI CD31
<0.0001
0.0257
<0.0001
0.0036
CD38-
CD38+
CD38++
J
FMO CD31
SP8 thymocytes
SP4 thymocytes
CD31-
CD31+
CD31++
FCS
CD31 : BV605
K
naive CD8+ T cells
naive CD4+ T cells
HELIOS : AF488
PTK7 : PE
CD31 : BV605
L
MFI HELIOS
MFI PTK7
0.0190
0.1888
0.5485
0.0118
0.1189
0.5863
0.5106
0.9203
0.7496
0.0249
0.6491
0.1716
CD31-
CD31+
CD31++
M
% of naive CD8+ T cells
% of naive CD4+ T cells
CD38-
CD38+
CD38++
CD31-
CD31+
CD31++
N
SSC-A
FSC-A
lymphocytes
FSC-W
single cells
FSC
T cells
CD3 : PE-Cy7
CD8+
CD4+
CD4 : APC-Cy7
Treg exclusion
FoxP3 : PB
naive CD4+
naive CD8+
CCR7 : APC
CD45RA : FITC

Supplementary Figure 2: Characterization of CD38 and CD31 expressing subsets of naive T cells, related to Figure 3.

(**A**) Violin plots showing scaled expression of CD38 antibody per cluster of $CD8^+$ and $CD4^+$ T cells.

(**B**) Representative cytometric gating strategy identifying naive T cells used for cell sorting.

(**C**) GSEA plots of SOX4+ signature of single-cell naive T cells or $CD38^{++}$ signature from sorted naive T cells enriched to sorted naive T cells.

(**D**) Absolute sjTREC content of sorted CD38 subsets of naive T cells.

(**E**) Representative cytometric gating strategy identifying naive T cells used for cohort flow analysis.

(**F**) Representative dot plots showing co-expression of CD38 and HELIOS or TOX of naive $CD8^+$ and $CD4^+$ T cells of a young donor.

(**G**) Representative histograms showing PTK7 expression by naive T cells and SP thymocytes.

(H) Representative dot plots showing co-expression of CD38 and PTK7 of naive $CD8^+$ and $CD4^+$ T cells of a young donor.

(**I**) MFI of CD31 expression in CD38 subsets of naive $CD8^+$ (top) and $CD4^+$ (bottom) T cells, p-adj values by one-way ANOVA with Tukey's multiple comparisons test (n=15).

(**J**) Representative dot plots of CD31 subset gating strategy of naive $CD8^+$ (top) and $CD4^+$ (bottom) T cells.

(**K**) Representative dot plots showing co-expression of CD31 and HELIOS or PTK7 of naive $CD8^+$ and $CD4^+$ T cells of a young donor.

(**L**) MFI of HELIOS and PTK7 expression in CD31 subsets of naive T cells, p-adj values by one-way ANOVA with Tukey's multiple comparisons test (n=15).

(**M**) Scatter plots showing the percentage of CD38 subsets and CD31 subsets of naive T cells (n=15).

(**N**) Representative cytometric gating strategy identifying naive T cells used for analysis of thymectomy cohort.

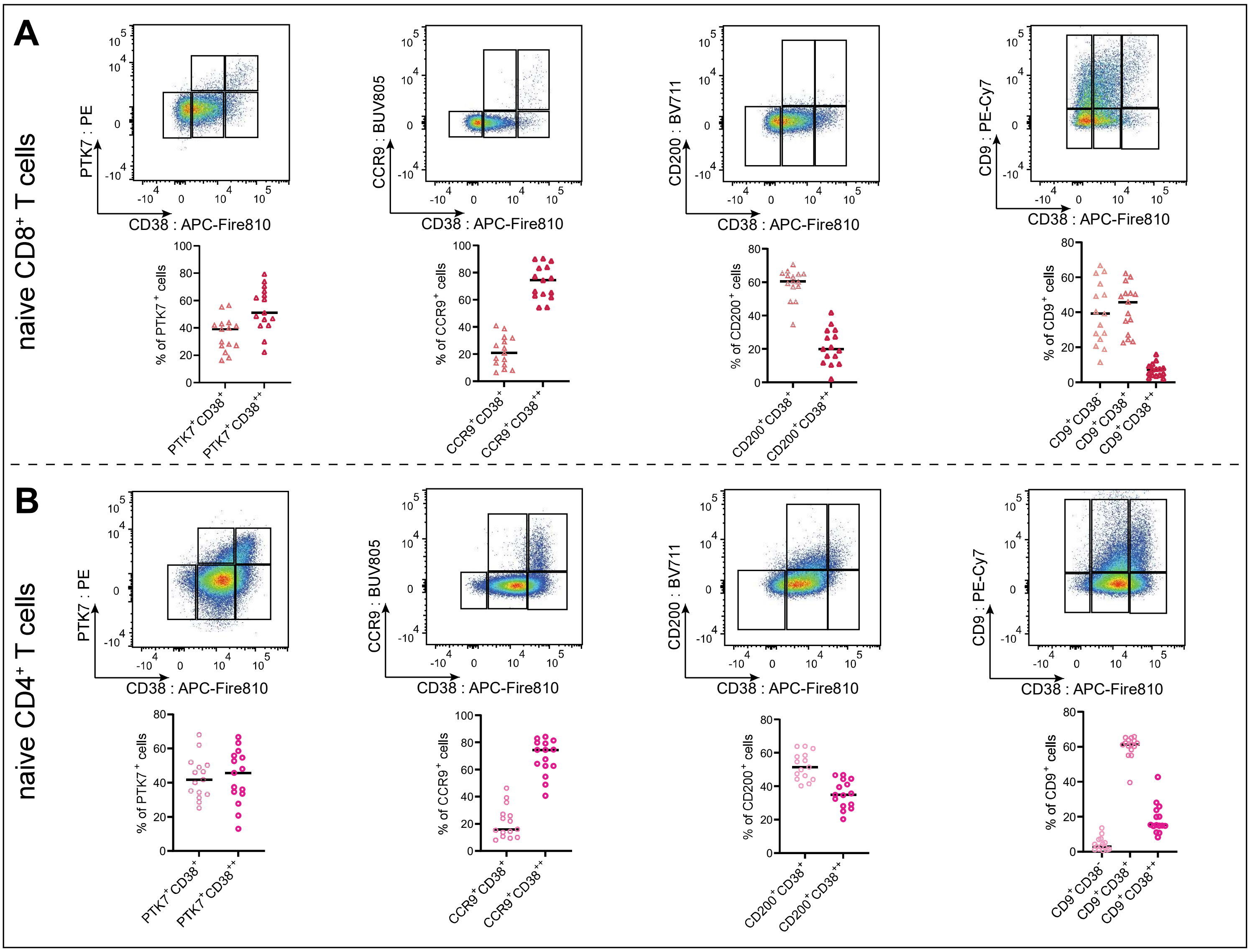
A
naive CD8+ T cells
PTK7 : PE
CCR9 : BUV805
CD200 : BV711
CD9 : PE-Cy7
CD38 : APC-Fire810
% of PTK7+ cells
% of CCR9+ cells
% of CD200+ cells
% of CD9+ cells
PTK7+CD38+
PTK7+CD38++
CCR9+CD38+
CCR9+CD38++
CD200+CD38+
CD200+CD38++
CD9+CD38-
CD9+CD38+
CD9+CD38++
B
naive CD4+ T cells

Supplementary Figure 3: Additional RTE markers gating, related to Figure 4

(**A**) Representative dot plots showing the gating of additional RTE markers in naive $CD8^+$ T cells in the context of CD38 expression based on negative (FMO CD38) and positive (neonatal thymocytes) control (top). Scatter plots showing the percentage of naive $CD8^+$ cells expressing the additional RTE marker associated with $CD38^+$ or $CD38^{++}$ cells within a population of RTE marker-expressing cells (bottom).

(**B**) Representative dot plots showing the gating of additional RTE markers in naive $CD4^+$ T cells in the context of CD38 expression based on negative (FMO CD38) and positive (neonatal thymocytes) control (top). Scatter plots showing the percentage of naive $CD4^+$ cells expressing the additional RTE marker associated with $CD38^+$ or $CD38^{++}$ cells within a population of RTE marker-expressing cells (bottom).

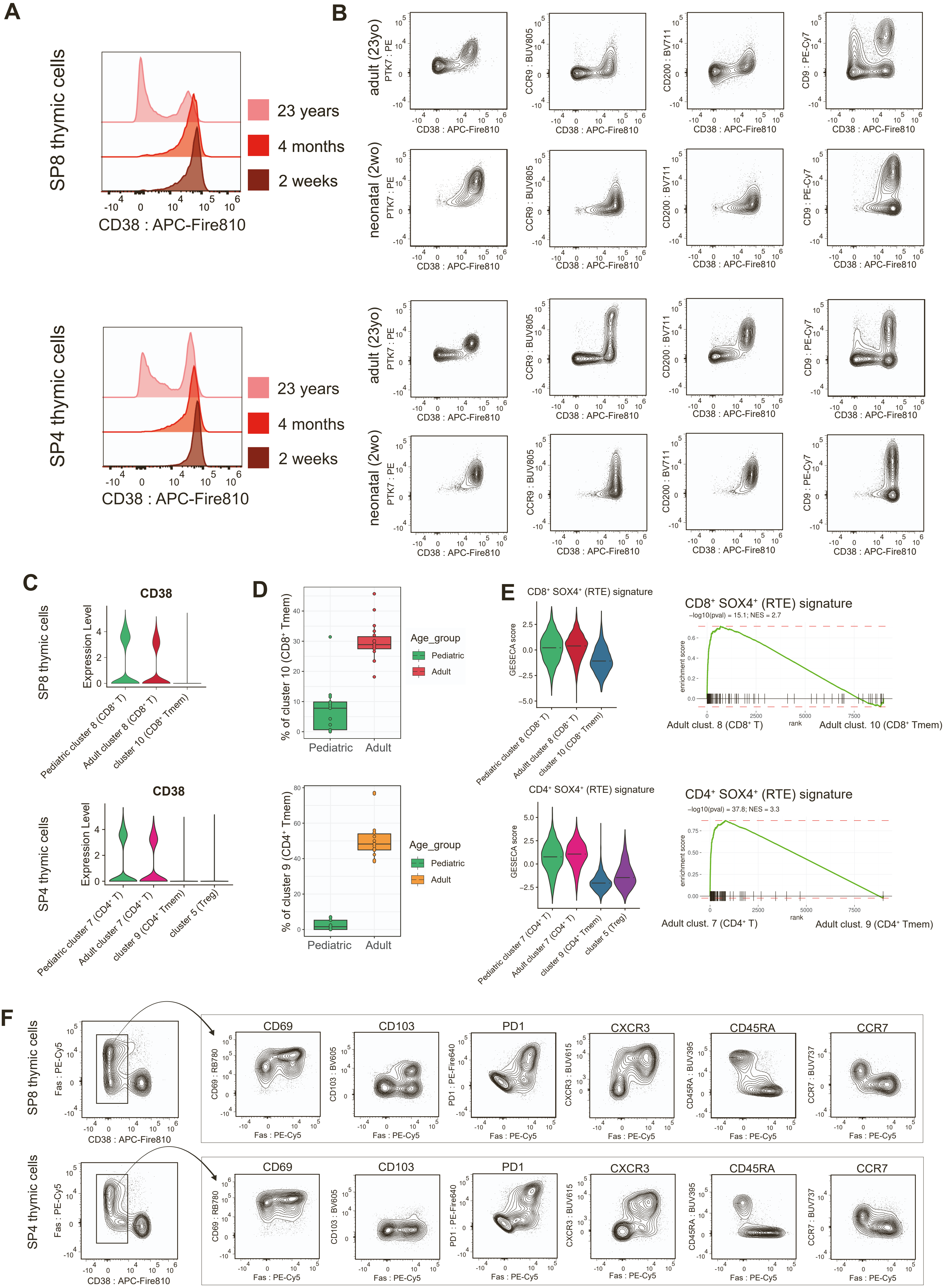
A
SP8 thymic cells
23 years
4 months
2 weeks
CD38 : APC-Fire810
SP4 thymic cells
B
adult (23yo)
neonatal (2wo)
PTK7 : PE
CCR9 : BUV805
CD200 : BV711
CD9 : PE-Cy7
C
CD38
Expression Level
Pediatric cluster 8 (CD8+ T)
Adult cluster 8 (CD8+ T)
cluster 10 (CD8+ Tmem)
Pediatric cluster 7 (CD4+ T)
Adult cluster 7 (CD4+ T)
cluster 9 (CD4+ Tmem)
cluster 5 (Treg)
D
% of cluster 10 (CD8+ Tmem)
% of cluster 9 (CD4+ Tmem)
Age_group
Pediatric
Adult
E
CD8+ SOX4+ (RTE) signature
−log10(pval) = 15.1; NES = 2.7
GESECA score
enrichment score
rank
Adult clust. 8 (CD8+ T)
Adult clust. 10 (CD8+ Tmem)
CD4+ SOX4+ (RTE) signature
−log10(pval) = 37.8; NES = 3.3
Adult clust. 7 (CD4+ T)
Adult clust. 9 (CD4+ Tmem)
F
Fas : PE-Cy5
CD69 : RB780
CD103 : BV605
PD1 : PE-Fire640
CXCR3 : BUV615
CD45RA : BUV395
CCR7 : BUV737
CD69
CD103
PD1
CXCR3
CD45RA
CCR7

Supplementary Figure 4: Phenotype analysis of SP thymic cells, related to Figure 4.

(**A**) Representative histograms showing CD38 expression of SP thymic cells in donors of different ages.

(**B**) Counterplots of RTE-related markers expression patterns of adult and neonatal thymic cells.

(**C**) Violin plots showing normalized expression of CD38 per cluster of thymic cells. Clustering and labels are retrieved from Park et al. [3] (Figure 1c).

(**D**) Boxplots showing the proportion of Tmem thymic cells within $CD8^+$/$CD4^+$ T and $CD8^+$/$CD4^+$ Tmem cell clusters between pediatric (n_{sample}=13) and adult (n_{sample}=18) donors. Clustering and labels are retrieved from Park et al. [3] (Figure 1c).

(**E**) Violin plots showing single-cell $SOX4^+$ cluster signature enrichment score for thymic cell clusters (left). GSEA plots of single-cell $CD4^+$ $SOX4^+$ cluster signature enriched to adult $CD8^+$/$CD4^+$ T clusters. Clustering and labels are retrieved from Park et al. [3] (Figure 1c).

(**F**) Counterplots showing expression patterns of selected molecules within the population of $CD38^-$ thymic cells of adult (23yo) donor.

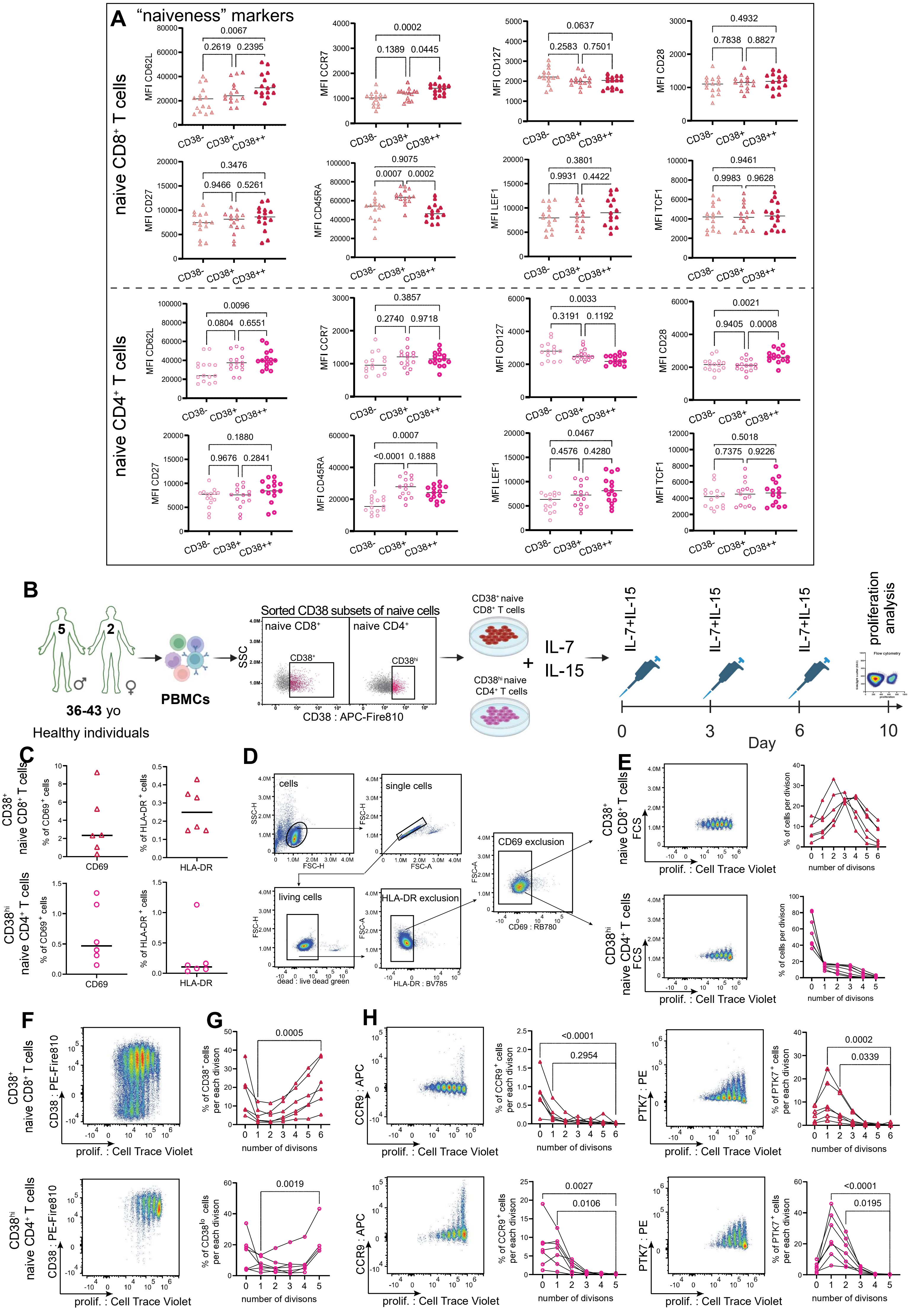

A
"naiveness" markers
naive CD8+ T cells
MFI CD62L
0.0067
0.2619
0.2395
MFI CCR7
0.0002
0.1389
0.0445
MFI CD127
0.0637
0.2583
0.7501
MFI CD28
0.4932
0.7838
0.8827
MFI CD27
0.3476
0.9466
0.5261
MFI CD45RA
0.9075
0.0007
0.0002
MFI LEF1
0.3801
0.9931
0.4422
MFI TCF1
0.9461
0.9983
0.9628
naive CD4+ T cells
MFI CD62L
0.0096
0.0804
0.6551
MFI CCR7
0.3857
0.2740
0.9718
MFI CD127
0.0033
0.3191
0.1192
MFI CD28
0.0021
0.9405
0.0008
MFI CD27
0.1880
0.9676
0.2841
MFI CD45RA
0.0007
<0.0001
0.1888
MFI LEF1
0.0467
0.4576
0.4280
MFI TCF1
0.5018
0.7375
0.9226
CD38-
CD38+
CD38++
B
5
2
♂
♀
36-43 yo
Healthy individuals
PBMCs
Sorted CD38 subsets of naive cells
naive CD8+
naive CD4+
SSC
CD38+
CD38hi
CD38 : APC-Fire810
CD38+ naive CD8+ T cells
CD38hi naive CD4+ T cells
IL-7
IL-15
IL-7+IL-15
proliferation analysis
Day
0
3
6
10
C
CD38+ naive CD8+ T cells
% of CD69+ cells
CD69
% of HLA-DR+ cells
HLA-DR
CD38hi naive CD4+ T cells
D
cells
single cells
living cells
HLA-DR exclusion
CD69 exclusion
SSC-H
FSC-H
FSC-A
dead : live dead green
HLA-DR : BV785
CD69 : RB780
E
CD38+ naive CD8+ T cells
CD38hi naive CD4+ T cells
FCS
prolif. : Cell Trace Violet
% of cells per divison
number of divisons
F
CD38+ naive CD8+ T cells
CD38hi naive CD4+ T cells
CD38 : PE-Fire810
prolif. : Cell Trace Violet
G
% of CD38+ cells per each divison
0.0005
% of CD38lo cells per each divison
0.0019
number of divisons
H
CCR9 : APC
% of CCR9+ cells per each divison
<0.0001
0.2954
0.0027
0.0106
PTK7 : PE
% of PTK7+ cells per each divison
0.0002
0.0339
<0.0001
0.0195
prolif. : Cell Trace Violet
number of divisons

Supplementary Figure 5: Additional phenotype analysis of $CD38^{++}$ RTEs, and RTE proliferation, related to Figure 4.

(**A**) MFI of selected naive T cell marker expression in $CD38^{-/+/++}$ naive T cells. p-adj values by one-way ANOVA with Tukey's multiple comparisons test (n=15)

(**B**) Scheme of experimental design of homeostatic proliferation *in vitro*.

(**C**) Scatter plots showing the percentage of HLA-DR^{+} and $CD69^{+}$ naive $CD8^{+}$ (top) and $CD4^{+}$ (bottom) T cells after 10-day incubation with IL-7 and IL-15.

(**D**) Representative cytometric gating strategy identifying HLA-DR^{-} and $CD69^{-}$ naive T cells used for homeostatic proliferation experiments.

(**E**) Representative dot plots showing naive T cell proliferation (left). Scatter plots showing the percentage of naive T cells per each cell division (n=7).

(**F**) Representative dot plots showing CD38 expression of naive T cell in the context of proliferation cycle.

(**G**) Scatter plots showing the percentage of $CD38^{-/lo}$ naive T cells per each cell division.

(**H**) Representative dot plots showing CCR9 and PTK7 expression of naive T cells in the context of proliferation cycle. Scatter plots showing the percentage of $CCR9^{+}$ and $PTK7^{+}$ naive T cells per each cell division. p-adj values by one-way repeated measures ANOVA with Tukey's multiple comparisons test (n=7).

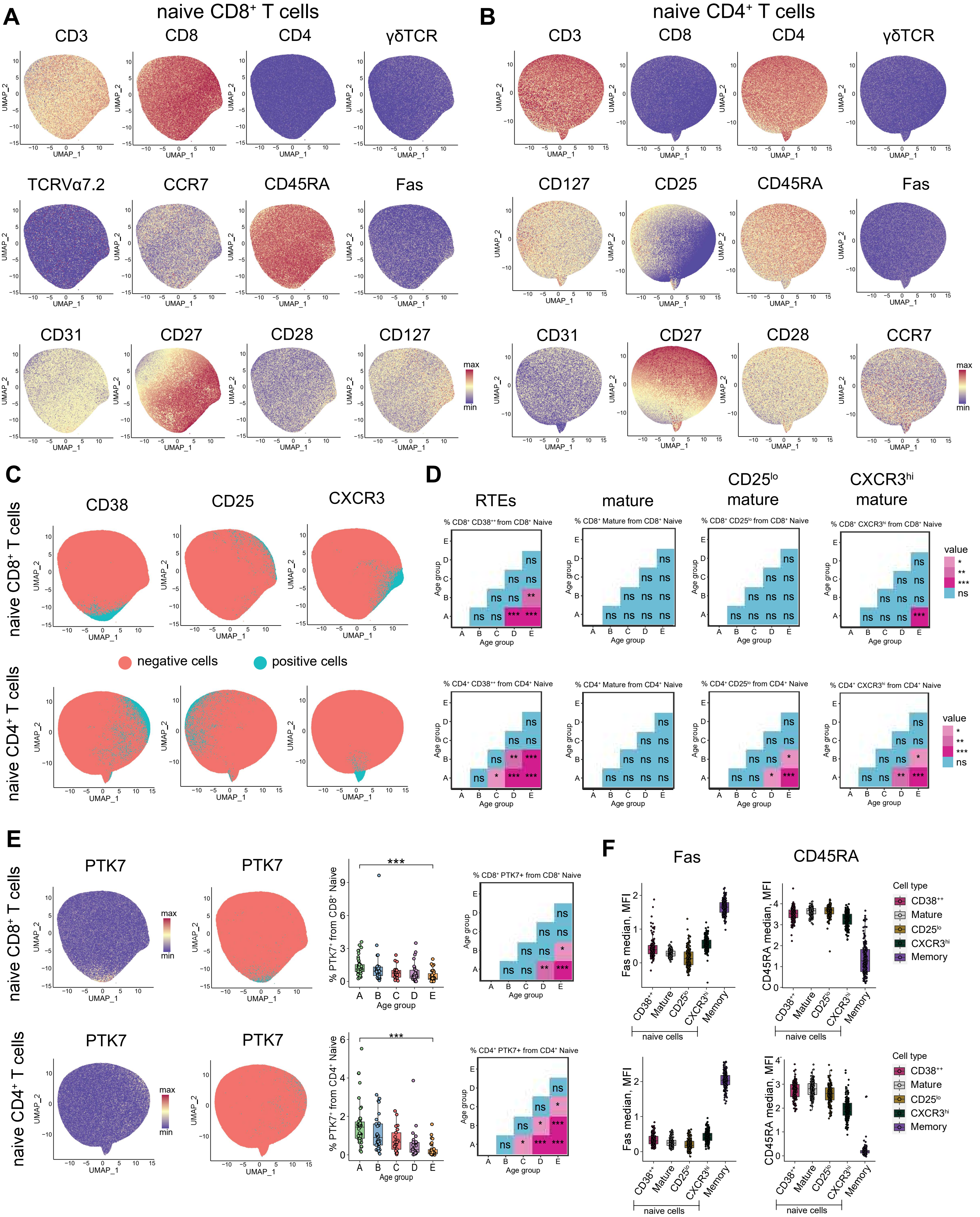

A
naive CD8+ T cells
CD3
CD8
CD4
γδTCR
TCRVα7.2
CCR7
CD45RA
Fas
CD31
CD27
CD28
CD127
B
naive CD4+ T cells
CD3
CD8
CD4
γδTCR
CD127
CD25
CD45RA
Fas
CD31
CD27
CD28
CCR7
max
min
UMAP_1
UMAP_2
C
naive CD8+ T cells
naive CD4+ T cells
CD38
CD25
CXCR3
negative cells
positive cells
D
RTEs
mature
CD25lo mature
CXCR3hi mature
% CD8+ CD38++ from CD8+ Naive
% CD8+ Mature from CD8+ Naive
% CD8+ CD25lo from CD8+ Naive
% CD8+ CXCR3hi from CD8+ Naive
% CD4+ CD38++ from CD4+ Naive
% CD4+ Mature from CD4+ Naive
% CD4+ CD25lo from CD4+ Naive
% CD4+ CXCR3hi from CD4+ Naive
Age group
value
*
**
***
ns
E
PTK7
% PTK7+ from CD8+ Naive
% PTK7+ from CD4+ Naive
% CD8+ PTK7+ from CD8+ Naive
% CD4+ PTK7+ from CD4+ Naive
F
Fas
CD45RA
Fas median, MFI
CD45RA median, MFI
Cell type
CD38++
Mature
CD25lo
CXCR3hi
Memory
naive cells

Supplementary Figure 6: Detailed characterization of naive T cells in the aging cohort, related to Figure 5.

(**A**) UMAP plots with a surface expression of selected markers of naive $CD8^+$ T cells.

(**B**) UMAP plots with a surface expression of selected markers of naive $CD4^+$ T cells.

(**C**) UMAP plots showing thresholds for CD38, CD25, and CXCR3 gating.

(**D**) Heatmaps representing significance for pairwise comparisons of naive T cell subset percentages between age groups A–E, p-adj by post hoc Dunn's test after one-way Kruskal-Wallis test with Holm correction method (n=158 overall (A=45, B=29, C=21, D=28, E=35)). p-adj values were additionally corrected with Bonferroni method by the number of subpopulations in the comparison.

(**E**) UMAP plots with a surface expression of PTK7 of naive T cells. UMAP plots showing thresholds for PTK7 gating. Boxplots showing the percentage of $PTK7^+$ naive $CD8^+$ and $CD4^+$ T cells by age groups A–E, p-adj by post hoc Dunn's test after one-way Kruskal-Wallis test with Holm correction method (n=158 overall (A=45, B=28, C=21, D=28, E=35)). p-adj values were additionally corrected with Bonferroni method by the number of subpopulations in the comparison.

(**F**) Box plots showing MFI of Fas and CD45RA expression by subsets of $CD8^+$ and $CD4^+$ T cells.

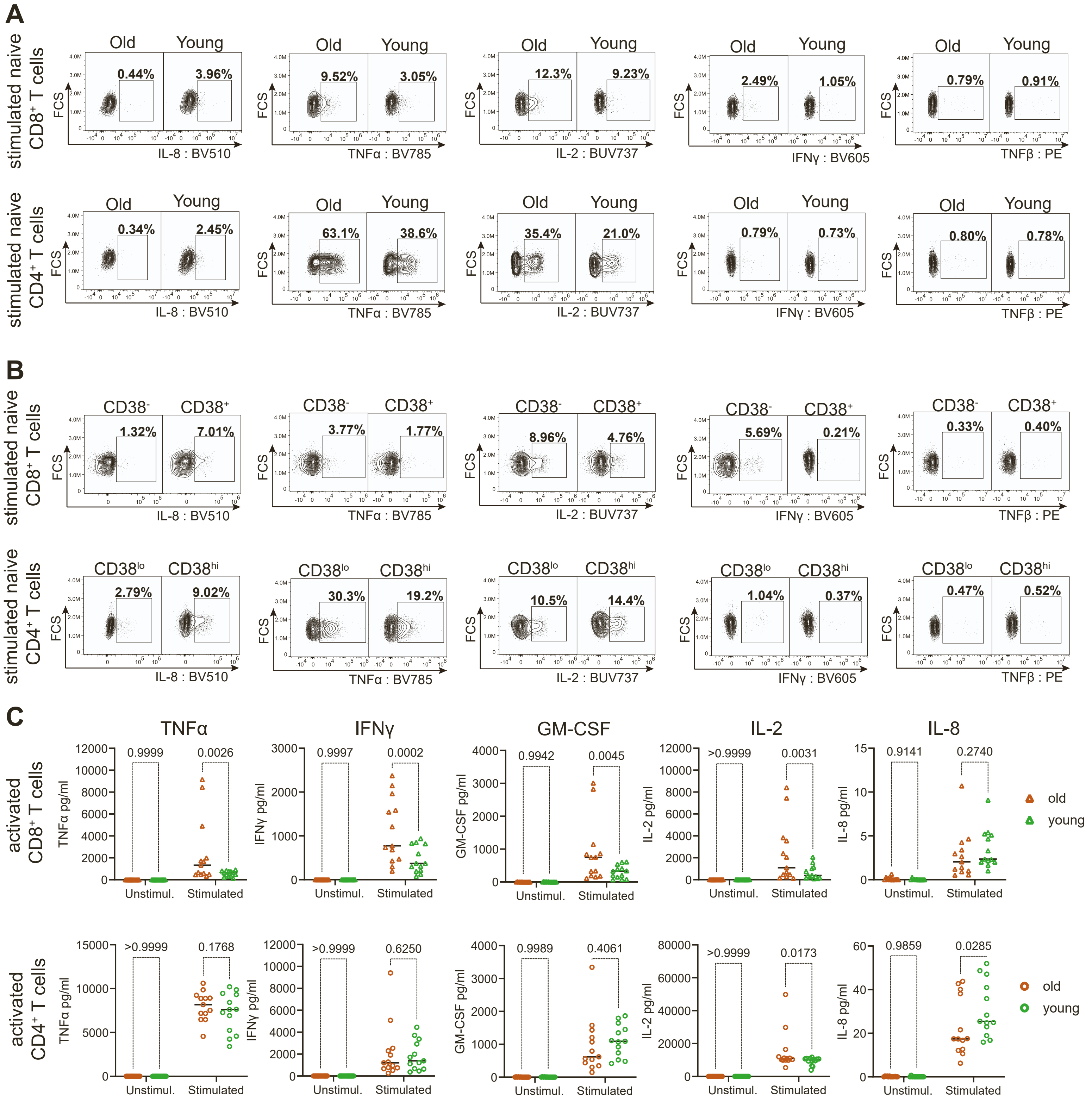

A
stimulated naive CD8+ T cells
stimulated naive CD4+ T cells
Old
Young
FCS
0.44%
3.96%
9.52%
3.05%
12.3%
9.23%
2.49%
1.05%
0.79%
0.91%
0.34%
2.45%
63.1%
38.6%
35.4%
21.0%
0.79%
0.73%
0.80%
0.78%
IL-8 : BV510
TNFα : BV785
IL-2 : BUV737
IFNγ : BV605
TNFβ : PE
B
CD38-
CD38+
CD38lo
CD38hi
1.32%
7.01%
3.77%
1.77%
8.96%
4.76%
5.69%
0.21%
0.33%
0.40%
2.79%
9.02%
30.3%
19.2%
10.5%
14.4%
1.04%
0.37%
0.47%
0.52%
C
activated CD8+ T cells
activated CD4+ T cells
TNFα
IFNγ
GM-CSF
IL-2
IL-8
TNFα pg/ml
IFNγ pg/ml
GM-CSF pg/ml
IL-2 pg/ml
IL-8 pg/ml
0.9999
0.0026
0.9997
0.0002
0.9942
0.0045
>0.9999
0.0031
0.9141
0.2740
>0.9999
0.1768
>0.9999
0.6250
0.9989
0.4061
>0.9999
0.0173
0.9859
0.0285
Unstimul.
Stimulated
old
young

Supplementary Figure 7: Representative gating of cytokine-positive naive T cells and cytokine production by naive T cell subsets, related to Figures 6, 7.

(**A**) Representative counterplots of cytokine expression of PMA/ionomycin-stimulated naive $CD8^+$ and $CD4^+$ T cells of young and old donors.

(**B**) Representative counterplots of cytokine expression of CD38 subsets of PMA/ionomycin-stimulated naive $CD8^+$ and $CD4^+$ T cells.

(**C**) Scatter plots showing cytokine production by $CD8^+$ (top) and $CD4^+$ (bottom) T cells of young (<35yo) and old (>64yo) in unstimulated and beads-activated conditions (n=13), p-adj values by two-way ANOVA with Tukey post-hoc.

References

1. Mogilenko, D.A., Shpynov, O., Andhey, P.S., Arthur, L., Swain, A., Esaulova, E., Brioschi, S., Shchukina, I., Kerndl, M., Bambouskova, M., et al. (2021). Comprehensive Profiling of an Aging Immune System Reveals Clonal GZMK+ CD8+ T Cells as Conserved Hallmark of Inflammaging. Immunity 54, 99-115.e12. https://doi.org/10.1016/j.immuni.2020.11.005.
2. Van Den Broek, T., Delemarre, E.M., Janssen, W.J.M., Nievelstein, R.A.J., Broen, J.C., Tesselaar, K., Borghans, J.A.M., Nieuwenhuis, E.E.S., Prakken, B.J., Mokry, M., et al. (2016). Neonatal thymectomy reveals differentiation and plasticity within human naive T cells. Journal of Clinical Investigation 126, 1126–1136. https://doi.org/10.1172/JCI84997.
3. Park, J.E., Botting, R.A., Conde, C.D., Popescu, D.M., Lavaert, M., Kunz, D.J., Goh, I., Stephenson, E., Ragazzini, R., Tuck, E., et al. (2020). A cell atlas of human thymic development defines T cell repertoire formation. Science (1979) 367. https://doi.org/10.1126/science.aay3224.
